# Discovery of Coerumycin, a Cinnamycin-like Lantibiotic from *Actinomadura coerulea* TMS085

**DOI:** 10.3390/antibiotics15010104

**Published:** 2026-01-21

**Authors:** Denis Iliasov, Thorsten Mascher

**Affiliations:** General Microbiology, TUD University of Technology Dresden, 01217 Dresden, Germany

**Keywords:** natural compounds, *Actinomadura* sp., *Bacillus subtilis*, cinnamycin, resistance development, whole-cell biosensors

## Abstract

**Background:** The current rise in multidrug-resistant pathogens highlights the urgent need for the discovery of novel antibacterial agents with potential clinical applications. A considerable proportion of these developed resistances may be attributable to the intrinsic response of bacteria to antibiotic-induced stress conditions in the environment. Consequently, the identification and characterization of genetic alterations in physiological processes in response to antibiotics represent promising strategies for the discovery and characterization of naturally produced novel antibacterial agents. This study investigated the antimicrobial activity of an antimicrobial active isolate *Actinomadura coerulea* derived from a meerkat fecal sample. **Methods:** The production of secondary metabolites that potentially compromise bacterial cell wall integrity was confirmed by the induction of promoter activity in whole-cell biosensors in which an antibiotic-inducible promoter was fused to the luciferase cassette. During plate-based biosensor assays, we identified naturally resistant *Bacillus subtilis* colonies growing in the zone of inhibition around *A. coerulea* colonies. After these successive rounds of selection, highly resistant spontaneous *B. subtilis* mutants had evolved that were subjected to whole-genome sequencing. **Results:** Non-silent mutations were identified in *pssA*, which encodes a phosphatidylserine synthase; *mdtR*, as a gene for the repressor of multidrug resistance proteins, and *yhbD*, whose function is still unknown. A new cinnamycin-like molecule, coerumycin, was discovered based on the physiological role of PssA and comprehensive genomic analysis of *A. coerulea*. Additional experiments with cell extracts containing coerumycin as well as the cinnamycin-like compound duramycin confirmed that the interaction between coerumycin and the bacterial cell envelope is inhibited by a loss-of-function mutation in *pssA*. **Conclusion**: Our approach demonstrates that combining the exploration of niche habitats for actinomycetes with whole-cell biosensor screening and characterization of natural resistance development provides a promising strategy for identifying novel antibiotics.

## 1. Introduction

Since their discovery in the 20th century, naturally occurring antibiotic substances have played essential roles in the treatment of countless bacterial infections. In light of the current global spread of multidrug-resistant strains and bacterial infections, the discovery and development of new antibiotics from natural sources are now of paramount importance [1]. Thus far, the chemical synthesis and modification of new and existing antibiotic substances for the control of infectious diseases have been employed. Nevertheless, the clinical application of these synthetic and semi-synthetic antibiotics is also accompanied by the rapid emergence of naturally resistant strains [2,3]. One potential avenue for accessing the novel antimicrobial diversity of the Earth’s biospheres is the isolation of strains, including actinomycetes, from unexplored habitats. The phylum *Actinomycetota* (or actinobacteria) represents a primary and historically important source of diverse bioactive secondary metabolites. This phylum contains heterotrophic and chemoautotrophic, predominantly aerobic bacteria that exhibit considerable variation in their morphological, physiological, and cytochemical properties [4,5,6]. The taxonomic diversity of these bacteria ranges from well-characterized and widely distributed *Streptomyces* spp. to more exotic and rare species. Access to these rare *Actinobacteria*, therefore, increases the chances of discovering new bioactive natural products [4,7]. Recent studies have demonstrated that *Streptomycetes* and other actinomycetes are well adapted to live in symbiosis with plants and invertebrates [8,9,10,11,12]. Moreover, the gut microbiome of vertebrates such as *Elephas maximus*, *Hylobates hoolock*, and *Giraffa camelopardalis* has been shown to contain several novel microbial species and natural antimicrobial compounds, thereby substantiating the need for animal gut microbiome characterization as a potential source of new antimicrobial-producing *Streptomyces* spp. and other endemic filamentous strains [13,14,15]. Apart from *Streptomyces* species, recent studies have also highlighted the role of lesser-known genera, including *Micromonospora* and *Streptosporangiales*, and *Amycolatopsis* as a source of antibacterial, antifungal, and anticarcinogenic therapeutics. Many clinically relevant antibiotics have been isolated from these rare *Actinomycetes*, such as rifamycin from *Amycolatopsis rifamycinica*, vancomycin from *Amycolatopsis orientalis*, and erythromycin from *Saccharopolyspora erythraea* [16,17,18]. Another promising member of the *Thermosporaceae* family is Gram-positive, filamentous *Actinomadura* species. Despite its initial isolation in 1968 and the production of highly potent antimicrobial compounds (e.g., polyether tetronate ionophore tetromadurin SF2487 from *A. verrucosospora*, matlystatins from *A. atramentaria* as inhibitors of type IV collagenases, and different madurastatins) with broad-range activity, the genus *Actinomadura* has remained vastly underexploited [19,20].

Due to these complex biological interactions between the microorganisms within a habitat, bacteria have developed a variety of cellular defense mechanisms to protect themselves from the inhibitory effects of these antibacterial compounds. One of the aspects of this response is the development of resistance via natural changes at genetic, physiological, and metabolic levels that enable microorganisms to survive under increased competition [21]. Some bacteria exhibit intrinsic resistance to specific classes of antibiotics, due to the absence of antibiotic targets (vancomycin resistance in lactobacilli) or specific metabolic pathways required for the activation and action of antibiotics (metronidazole resistance in aerobic bacteria) [22,23,24]. In addition to natural resistance mechanisms based on active export of antibiotics by efflux pumps and the production of antibiotic-degrading or -modifying enzymes (such as the β-lactamase activity of *Staphylococcus aureus* [25]), mutation-based resistance mechanisms have also been identified. Here, analyzing the vertical (or endogenous) evolution of bacteria is a promising strategy for tracking the development of resistance. Random chromosomal mutations of a few base pairs can occur during bacterial replication, which can lead to the replacement of one or a few amino acids in a critical target (enzyme, cell wall, or cell structure). This can, in turn, lead to, e.g., masking of the target molecule by antibiotic substances or to increased expression of natural resistance genes. In this process, an initial mutation conferring a survival advantage is often maintained by the selective pressure of antibiotic treatment, followed by further mutations conferring additional survival advantages. This process is, therefore, often gradual over several generations of bacterial subpopulations, and the cumulative effects on resistance are often evident [26]. In principle, the identification and characterization of natural resistance mechanisms and their dynamic development are important approaches to further exploiting the effects of antibiotic substances and, ultimately, finding possible solutions to the resistance crisis. These strategies have led to the development of antibiotic-resistance breakers as an alternative treatment. These compounds negatively affect bacterial resistance mechanisms, thereby increasing the activity and efficacy of known antibiotics (e.g., “Co-Amoxiclav” as a combination of amoxicillin as a β-lactam and clavulanic acid as a β-lactamase inhibitor) [22,27,28].

In this study, we investigate an antibiotic-producing actinobacterium isolated from a meerkat (*Suricata suricatta*) fecal sample with regard to its antimicrobial activity. The isolated strain was identified through genome analysis as *Actinomadura coerulea* and screened for the production of antibacterial and antifungal compounds using inhibition tests with multiple pro- and eukaryotic microorganisms. The isolated strain was found to possess a pronounced antibacterial effect against Gram-positive bacteria. This effect could be attributed to the production of secondary metabolites that disrupt the integrity of the cell wall or cell membrane. During plate-based biosensor assays, we identified naturally resistant *Bacillus subtilis* colonies growing in the zone of inhibition around *A. coerulea* colonies. After repeated rounds of selection, highly resistant, spontaneous *B. subtilis* mutants have evolved, which were subjected to whole-genome sequencing. Genomic mutations were identified that explained resistance mechanisms and provided a hint for the nature of the antibiotics produced by *A. coerulea*. Genome mining identified the BGC for a novel cinnamycin analog, coerumycin, which was further analyzed. The physiological role of these spontaneous mutations for resistance development and the mechanism of action of this peptide antibiotic were investigated through experiments utilizing the cinnamycin analog duramycin and coerumycin-containing culture supernatants of *A. coerulea*.

## 2. Results

### 2.1. Characterization and Phylogenetic Identification of Isolated Actinomycetes sp.

**Figure 1:**
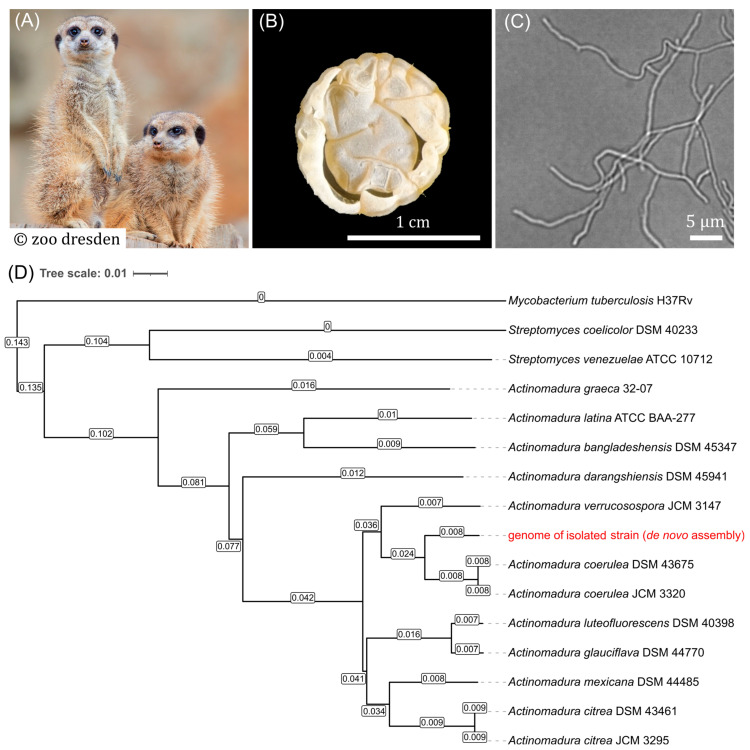
Colony and cellular morphology of the isolated *Actinomadura coerulea* strain. (**A**) The meerkats (*Suricata suricatta*). (**B**) Colony is depicted at day 10 of growth on MYM agar. Photos were taken with a P.CAM360 (2× magnification). (**C**) Microscopy pictures were obtained from liquid cultures of the isolates in MYM medium. Photos were taken with an exposure time of 3.6 s and 1000× magnification using an Axio Observer 7 inverse microscope (Carl Zeiss AG, Jena, Germany). The respective scale bars indicate 1 cm or 5 µm. (**D**) Phylogenetic tree containing the isolated strain. Tree inferred with FastME 2.1.6.1 from GBDP distances calculated from genome sequences [32]. Branch lengths are scaled in terms of GBDP distance formula d5. Numbers on branches denote GBDP pseudo-bootstrap support values >60% from 100 replications, with an average branch support of 84.9%. Tree is rooted at the midpoint and optimized using iTOL [33]. The phylogenetic classification of the isolated strain is highlighted in red.

The meerkat (*Suricata suricatta*) fecal sample, collected recently, was utilized for the isolation of actinobacteria. Following phenol treatment, dilution, and spreading of the fecal sample, an actinobacterium was isolated based on its characteristic colony and cell morphology. Macro- and microscopic observations of the isolate confirmed the presence of filamentous cells with branching, intricate cellular differentiation, including aerial hyphae formation, and sporulation, with hyphae measuring approximately 0.5–1 µm in diameter (Figure 1).

To classify the isolated strain phylogenetically, genomic DNA was sequenced using Sanger/Illumina paired-end sequencing and a quality control assessment was performed on the de novo assembled genome using the BUSCO 5.5.0 tool. The genome exhibited a guanine and cytosine (GC) content of 72.5% as well as both a high percentage of complete genes (96%) and low percentages of fragmented and missing genes (1.9% and 2.1%), which collectively indicate the high quality of the genome assembly [29]. The isolated strain was identified as belonging to the species group *Actinomadura coerulea* through phylogenetic characterization using the Type Strain Genome Server, TYGS (Figure 1D) [30]. The most closely related strain, *A. coerulea* DSM 43675 (GenBank accession GCF_014208105.1), was also identified by Similar Genome Finder services (Mash distance < 0.05; dDDH > 70%; d6 = 81.5) and the de novo assembled genome from the isolated strain was aligned with the reference genome using FastANI [31]. The average nucleotide identity (ANI) of the whole genome between the two strains was 96.5%, indicating that the isolated strain belongs to the species *Actinomadura coerulea* [32,33].

**Figure A1:**
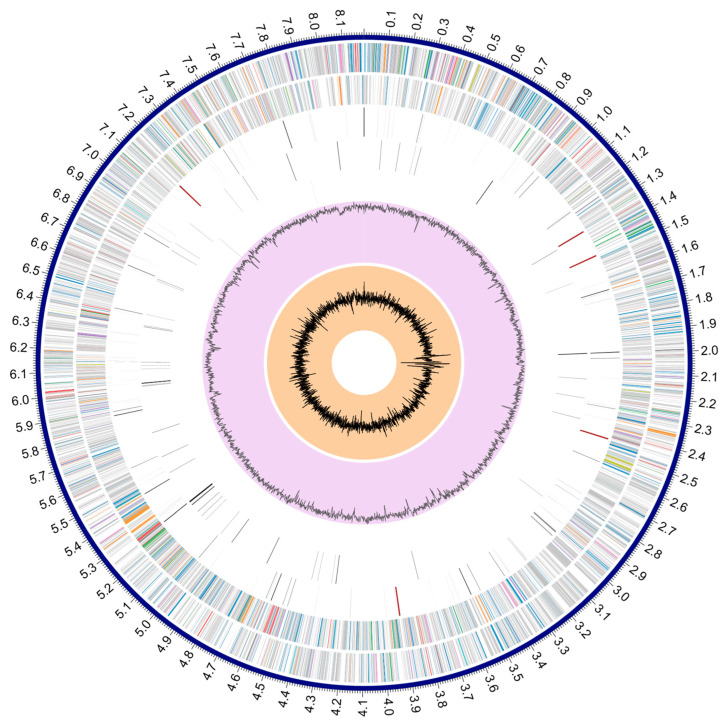
Circular graphical displays of the distribution of genome annotations and their subsystems for *A. coerulea* TMS085. Rings display (outwards to inwards): contigs (dark blue), CDSs on forward strand, CDSs on reverse strand, RNA genes, CDSs with homology to known antimicrobial resistance genes, CDSs with homology to known virulence factors, GC content (light purple ring), and GC skew (light orange ring). A subsystem is a set of proteins that together implement a specific biological process or structural complex and PATRIC annotation includes an analysis of the subsystems unique to genome. Band colors indicate the subsystems listed in the corresponding pie charts for strain (see Supplementary Materials S1).

The assembled genome of the isolate (*A. coerulea* TMS085) had a size of 8,188,457 bp (7908 total coding sequences, CDSs) (Figure A1) and was characterized using the comprehensive genome analysis tool from the Bacterial and Viral Bioinformatics Research Center (BV-BRC) [34,35]. A comparative analysis of the genome of the strain TMS085 and that of *A. coerulea* DSM 43675 revealed a higher number of genes involved in metabolic processes (972), DNA processing (105), cell envelope (21), and regulation and cell signaling (59) in the former (Table 1). Despite this, additional genes for cellular processes (4 additional genes) and membrane transport (11) were identified in the reference strain. The genome of strain TMS085 exhibited a high degree of similarity to the reference strain. However, several genomic variations (see Supplementary Materials Dataset S1) indicate that the isolate comprises a new *A. coerulea* strain.

**Figure A2:**
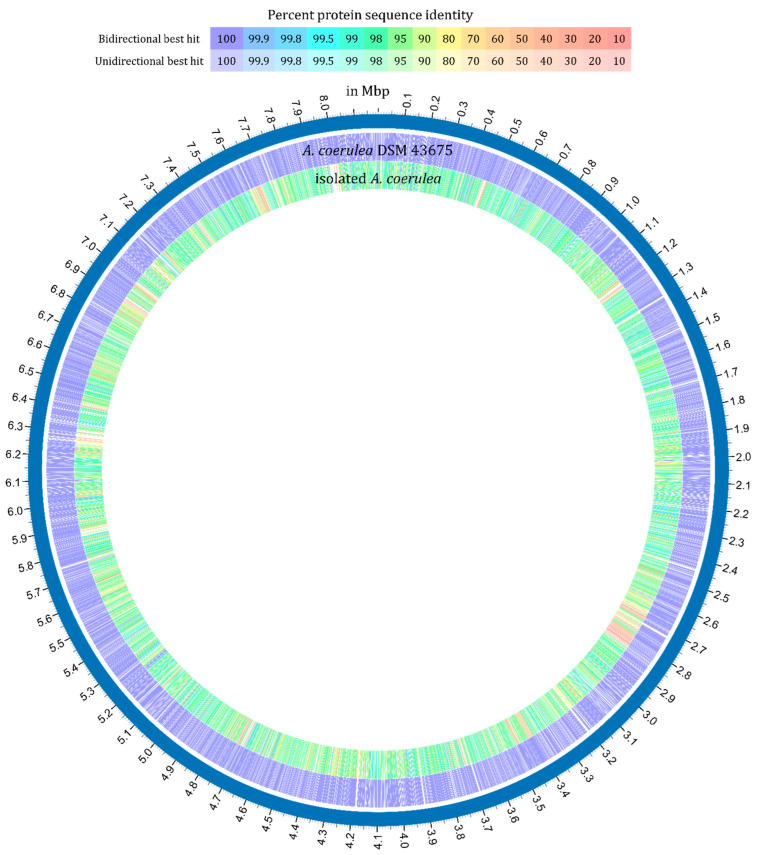
BV-BRC proteome comparison of *A. coerulea* TMS085 to the *A. coerulea* DSM 43675. Sequence identity between the *A. coerulea* and reference strains (from outside ring inwards: position in the genome, *A. coerulea* DSM 43675 to *A. coerulea* DSM 43675, and the isolated *A. coerulea* to *A. coerulea* DSM 43675). Color gradient corresponds to the percent protein sequence identity between the compared strains.

By aligning the genomes using snippy software in GalaxyEurope [36], a total of 141,833 nucleotide variations distributed throughout the genome were identified (see Supplementary Materials Dataset S1). These comprised single- and multiple-nucleotide polymorphisms (SNPs and MNPs or COMPLEX), nucleotide insertions (INS), or deletions (DEL) in both coding and non-coding regions. 97.8% of the variations (138,961) were assigned to single- and multiple-nucleotide polymorphisms. Additionally, a comparison of protein sequence identity was conducted using the BV-BRC web-based proteome comparison tool [34,35]. Compared to *A. coerulea* DSM 43675, less than 81% of the protein sequences identified in the genome of TMS085 were found to have over 95% sequence identity with the reference strain (Figure A2 and Supplementary Materials Dataset S2). Furthermore, only 45.6% of all proteins exhibit 98% similarity to the reference proteins. Although the isolated strain TMS085 is phylogenetically closely related to the reference strain, these genomic differences suggest that the isolate is a novel *A. coerulea* strain. The genome of strain TMS085 has been deposited in GenBank under the accession number PRJNA1365823 and is available in Supplementary Materials Dataset S1.

### 2.2. Production of Cell Envelope Antibiotics by Isolated A. coerulea TMS085

**Figure A3:**
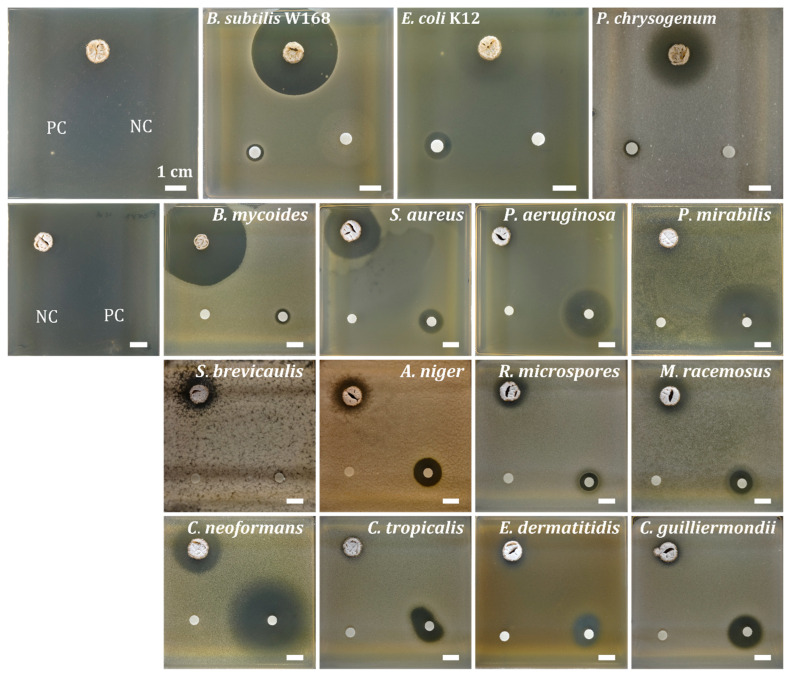
Antimicrobial activity of *A. coerulea* TMS085 against wild-type and laboratory bacterial and fungal strains. Following overnight incubation at 37 °C for bacterial strains and 28 °C for yeasts and fungi, inhibition of the strains tested by the iso-lates was photographically documented using a P.CAM360 (1.48× magnification), and radii of the zones of inhibition (ZOI) were measured (A). Scale bars indicate 1 cm. Positive (PC) and negative (NC) controls in the form of sterile filter discs soaked in 10 µL of the corresponding control were applied following spread plating. A 50% (*v*/*v*) glycerol solution functioned as the negative control for all overlays. Nisin (40 mg/mL) was a positive control for all Gram-positive bacterial strains (*B. subtilis* W168*, B. mycoides*, *S. aureus*) and norfloxacin (100 µg/mL)—for Gram-negative strains (*E. coli* K12, *P. aeruginosa*, *P. mirabilis*). For the fungi and yeasts, amphotericin B (250 µg/mL) served as a positive control. Cycloheximide (1 mg/mL) was used as a positive control for *C. neoformans*.

One of the most salient characteristics of actinomycetes is their capacity to produce secondary metabolites with antimicrobial activity [37]. The compounds produced by the isolated *A. coerulea* strain were initially evaluated against a panel of bacterial, yeast, and fungal strains including *Bacillus subtilis* W168, *Escherichia coli* K12, *Saccharomyces cerevisiae*, and *Penicillium chrysogenum*. Subsequently, four pathogenic bacterial strains and eight pathogenic fungal strains were also challenged with strain TMS085 to further characterize the activity spectra of the produced antimicrobial compounds (Figure A3).

**Figure 2:**
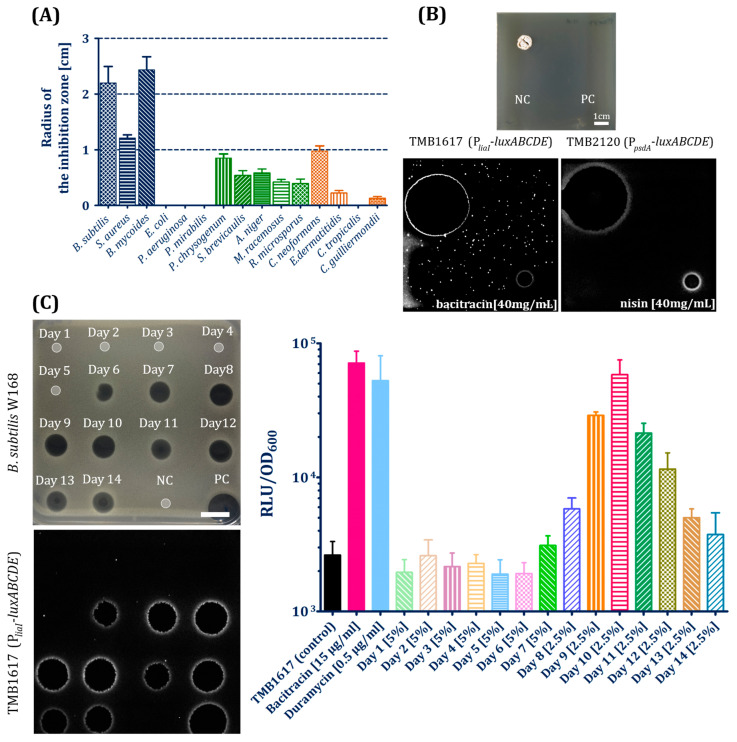
(**A**) Antimicrobial activity of *A. coerulea* TMS085 against wild-type and laboratory bacterial and fungal strains. Average radii of the measured ZOI against bacterial strains and yeasts were calculated and are displayed as bar graph with standard deviation. The absence of bars indicates no inhibition against the corresponding strain. (**B**) Screening of *A. coerulea* with the biosensor strains TMB1617 and TMB2120. Following isolate cultivation and spread plating of the biosensor strain, bioluminescence was measured (1 min exposure time). A 50% (*v*/*v*) glycerol solution functioned as the negative control (NC) for all overlays. The corresponding antibiotics were used as positive controls (PC; nisin [40 mg/mL] for TMB2120, bacitracin [40 mg/mL] for TMB1617). (**C**) Production of antimicrobial compounds in liquid culture by *A. coerulea*. Bioluminescence was measured (1 min exposure time). Bacitracin [50 mg/mL] was used as positive controls (PC). Sterile MYM medium was used as a negative control (NC). Numbers in the spotting layout square refer to the cultivation day. Scale bar indicates 1 cm. Quantification of biosensor promoter induction by the isolate supernatants was performed in plate reader assays measuring bacterial growth (as optical density at 600 nm (OD_600_)) and luminescence of the biosensors. Induction via the addition of supernatants or controls occurred at time point 60 min. Average relative luminescence units (RLU) and their standard deviations are depicted for TMB1617 to the time point 20 min post-induction (80 min). Controls consisted of the uninduced biosensor with the addition of 5 µL sterile LB. Bacitracin (in the final concentration per well 15 µg/mL) and duramycin (0.5 µg/mL) were used as the positive control. Plate reader assays were performed in biological and technical triplicates for each strain and cultivation time point.

**Figure A4:**
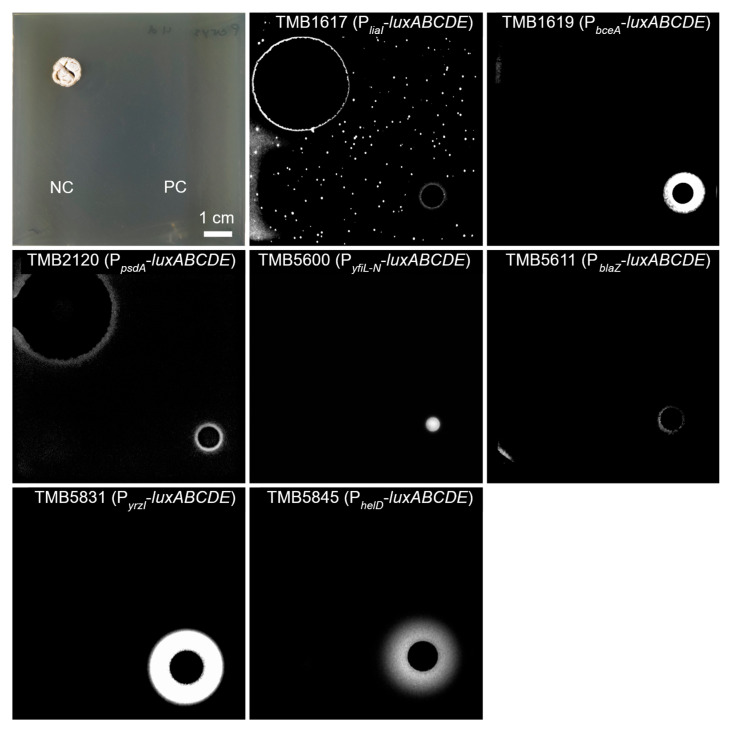
Screening of *A. coerulea* TMS085 with the biosensor strains. Following isolate cultivation and spread plating of the biosensor strain, bioluminescence was measured (1 min exposure time). A 50% (*v*/*v*) glycerol solution functioned as the negative control (NC) for all overlays. The corresponding antibiotics were used as positive controls (Table 3).

**Figure A5:**
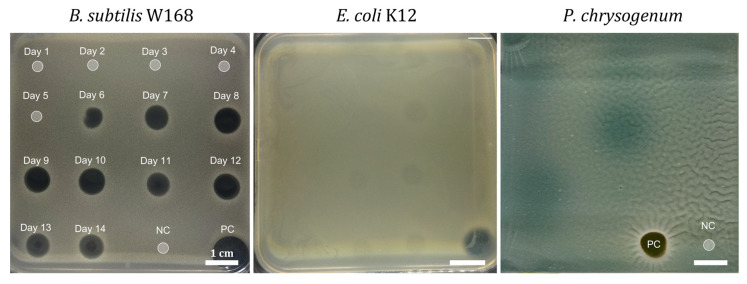
Production of antimicrobial compounds in liquid culture by *A. coerulea* TMS085. Following pour spreading, 10 µL of the sterile filtered supernatants or controls were spotted on top of the soft agar inoculated with *B. subtilis* W168, *E. coli* K12 and *P. chrysogenum*. Nisin (40 mg/mL) was a positive control for *B. subtilis* W168 and Norfloxacin (100 µg/mL)—for *E. coli* K12. For *P. chrysogenum*, amphotericin B (250 µg/mL) served as a positive control (PC). Sterile MYM medium was used as a negative control (NC). Numbers in the spotting layout square refer to the cultivation day. Scale bar indicates 1 cm.

Strain TMS085 displayed both antibacterial and antifungal activity to varying degrees. Moderately strong inhibition of Gram-positive bacteria (zone of inhibition (ZOI) > 1 cm) was observed for both laboratory and pathogenic strains including *Bacillus mycoides* from the *B. cereus* group and *Staphylococcus aureus* (Figure 2A and Figure A3). The production of antifungal substances was also observed as moderate activity against *P. chrysogenum* and other pathogenic fungal strains (ZOI < 1 cm). *A. coerulea* TMS085 displayed moderately high activity against *Cryptococcus neoformans* (ZOI approx. 1 cm), while no or only minimal activity was observed against all other pathogenic fungal and yeast strains (Figure A4 and Figure A5). As an opportunistic fungal pathogen, *C. neoformans* exhibits increased resistance to a number of clinically approved antimycotics, which presents a challenge to the successful drug treatment of diseases such as cryptococcal pneumonia, meningitis, and cryptococcemia [38].

To further elucidate the properties of the antimicrobial compounds produced by the natural isolates, a panel of whole-cell biosensors was implemented. These modified *Bacillus subtilis* W168 strains contain the *luxABCDE* operon for luminescence fused to an inducible antibiotic-specific promoter. The biosensors (TMB1617 and TMB2120) for compounds that compromise cell envelope integrity were induced by metabolites of strain TMS085 (Figure 2B). Induction of the P*_liaI_* biosensor (TMB1617) indicates the production of antimicrobial peptides that interfere with the lipid II cycle of cell wall biosynthesis, such as bacitracin, daptomycin, lantibiotics, ramoplanin, rhamnolipids, vancomycin, and cationic antimicrobial peptides (Figure 2B) [39]. Furthermore, the P*_psdA_* biosensor (TMB2120) was induced, which reacts specifically to lantibiotics, e.g., nisin (Figure 2B) [40,41].

**Figure A6:**
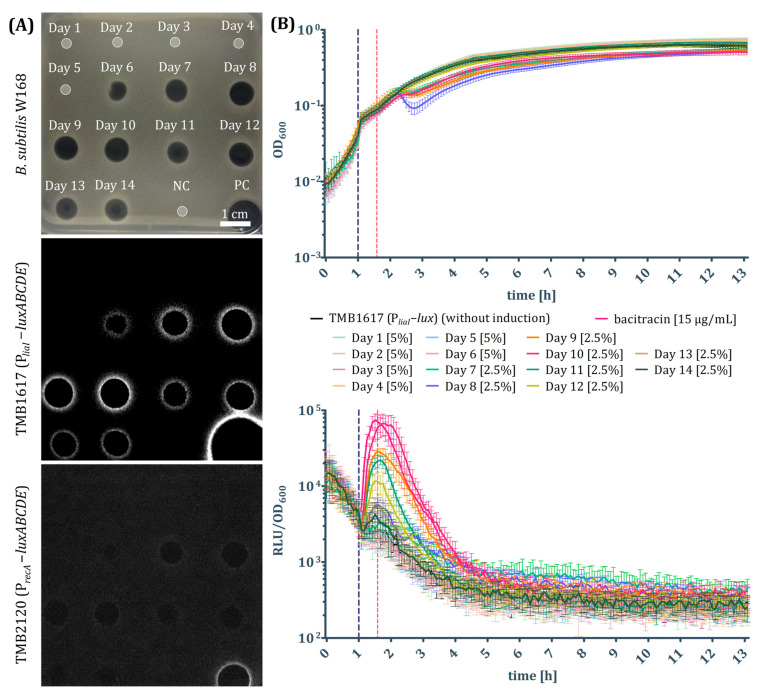
Production of antimicrobial compounds in liquid culture by *A. coerulea* TMS085 (**A**). Following pour spreading, 10 µL of the sterile filtered supernatants or controls were spotted on top of the soft agar inoculated with *B. subtilis* W168, TMB11617 and TMB2120. After incubation, inhibition was documented photographically with a P.CAM360 (1.48× magnification), and bioluminescence was measured (1 min exposure time). The corresponding antibiotics were used as positive controls (PC; nisin [40 mg/mL] for *B. subtilis* W168 and TMB2120 and bacitracin [50 mg/mL] for TMB1617). Sterile MYM medium was used as a negative control (NC). Numbers in the spotting layout square refer to the cultivation day. Scale bar indicates 1 cm. Growth and luminescence curves for biosensor TMB1617 testing of isolated *A. coerulea* TMS085 (**B**). RLU/OD_600_ values were generated in plate reader assays measuring optical density and luminescence in 5-min intervals over the course of 13 h. Supernatant samples are identified by time point (Day 1–14). The uninduced biosensor served as a negative control. Bacitracin (final concentration in well 15 μg/mL) was used as a positive control. Addition of 5 μL of the supernatants to the biosensor (=induction) is indicated at time point 1 h (dashed blue line). RLU averages and their standard deviations were calculated for each strain and cultivation day at the approximate peak of induction (20 min post-induction, dashed red line).

A temporal characterization of the antibacterial metabolites produced by *A. coerulea* TMS085 in liquid culture revealed the induction of the P*_liaI_*-*luxABCDE* biosensor (TMB1617) from the seventh day until the end of cultivation (Figure 2C). These findings correlate well with the previous results regarding the antimicrobial activity of the supernatants. Surprisingly, no activity of the P*_psdA_* promoter in TMB2120 was detected when challenged with supernatants (Figure A6). The P*_liaI_* biosensor bioluminescence was quantified for the supernatants from the cultures of *A. coerulea* TMS085 in plate reader assays. The strain TMS085 was cultivated in the liquid medium over 14 days. Supernatants were extracted daily and sterile filtered. The biosensor assays were performed using the sterile supernatants in sub-inhibitory concentrations (5% (*v*/*v*) of plate-reader-well for days one to six or 2.5% (*v*/*v*) of plate-reader-well for days eight to fourteen, as the concentrations that showed no negative effects on the growth of the biosensors during the experiments). The induction was successfully detected by the addition of the seven-day supernatant, which also correlates with the results of the spot-on-lawn experiments. This can be attributed to the activation of secondary metabolism under nutrient limitation at this time point. Similar to the observed increase in antibacterial activity of the supernatants, RLU values increased until the tenth day of cultivation. The average maximum RLU values of TMB1617 corresponded to approx. 22-fold increases in biosensor luminescence in comparison to the uninduced biosensor and were comparable to the positive control bacitracin (Figure 2C). From day ten onwards, biosensor promoter (P*_liaI_*) induction continuously declined, resulting in a 16-fold decrease in induction.

### 2.3. Development of Natural Resistance to Antibiotic Substances Produced by A. coerulea in B. subtilis

**Figure 3:**
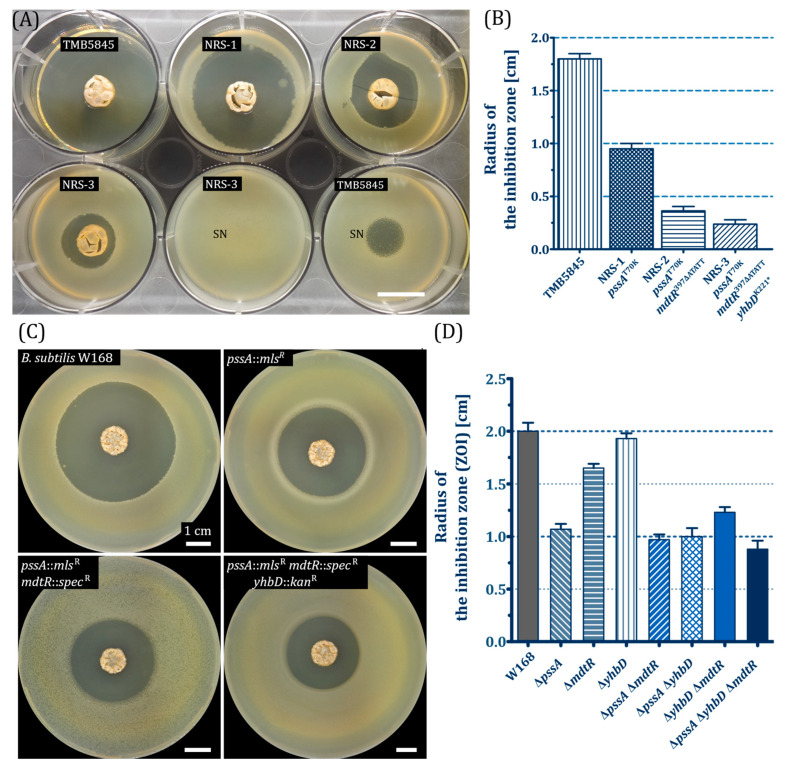
(**A**,**B**) Antimicrobial activity against resistance development *B. subtilis* strains. (**A**) Inhibition was documented photographically using a P.CAM360 (1.24× magnification), and inhibition was measured. Scale bar indicates 1 cm. 10 µL of sterile supernatant of eleven days cultivated *A. coerulea* (SN) was also used for the spot-on-lawn-assay with *B. subtilis* TMB5845 and NRB-3 (TMB5845 *pssA*^T70K^, *mdtR*^397∆ATATT^, *yhbD*^K221*^). (**B**) Averages of the radii of the ZOI were calculated and are displayed as a bar graph with their standard deviations. (**C**,**D**) Antimicrobial activity against *B. subtilis* mutants. (**C**) Inhibition was documented photographically using a P.CAM360 (1.48× magnification), and inhibition was measured. Scale bars indicate 1 cm. (**D**) Averages of the radii of the ZOI were calculated and are displayed as a bar graph with their standard deviations.

During the biosensor assays, we observed the growth of individual colonies in the zone of inhibition (ZOI). These spontaneous resistant colonies (NRS-1) were isolated and reduced activity of the metabolites produced by *A. coerulea* was confirmed (ZOI approx. 9 mm compared to 17 mm of the sensitive wild type (TMB5845)); the resulting ZOI again contained resistant colonies. Subsequent overlay experiments with these novel isolated strains (NRS-2) confirmed the further development of resistance to the antimicrobial compounds of *A. coerulea* (ZOI approx. 4 mm). Further directional selection of resistant *B. subtilis* colonies allowed for the isolation of strains exhibiting minimal growth inhibition by the antibiotics of *A. coerulea* (NRS-3; ZOI < 2.5 mm). Furthermore, the supernatant of an eleven-day *A. coerulea* culture did not affect the growth of these resistant strains (NRS-3) compared to the sensitive wild type (TMB5845; Figure 3A). The observed increase in resistance over multiple generations of the bacterial subpopulations indicates the cumulative effects of potential genomic mutations in the isolated strains.

Whole-genome sequencing was conducted on the isolated strains and the reference strain TMB5845 to identify the genetic mutations responsible for the development of resistance. Genomes were assembled and aligned using the alignment tool Snippy in the Galaxy platform (see Supplementary Materials Dataset S3). The alignment revealed a high percentage of nucleotide similarity between all three strains. Three novel non-silent mutations were identified in three genes (*pssA*, *mdtR*, and *yhbD*) in the resistance strains. The *pssA* mutation (C209A) was identified in the first resistance strain (NRS-1) and was retained through further selection. The detected single-nucleotide polymorphism (SNP) caused the threonine (T) at position 70 to be exchanged for a lysine (L). Furthermore, the same *mdtR* mutation was also detected in strains NRS-2 and NRS-3. A significant alteration in the protein sequence was identified through the deletion of five nucleotides (397ATATT). Additionally, the resistance strain NRS-3 encodes a non-silent nonsense mutation in *yhbD* (K221*), a component of the *yhbDEF* operon. For further characterization of the role of the natural non-silent mutation in *pssA* in the development of potential resistance to cell envelope antibiotics, single colonies of strain TMB1617 (NRS-4) were selected based on their ability to grow in the inhibition zone observed in the overlay experiments. The non-silent mutation in PssA was also identified by whole-genome sequencing, which may have a negative effect on the functionality of this protein (see Supplementary Materials Dataset S3).

**Figure A7:**
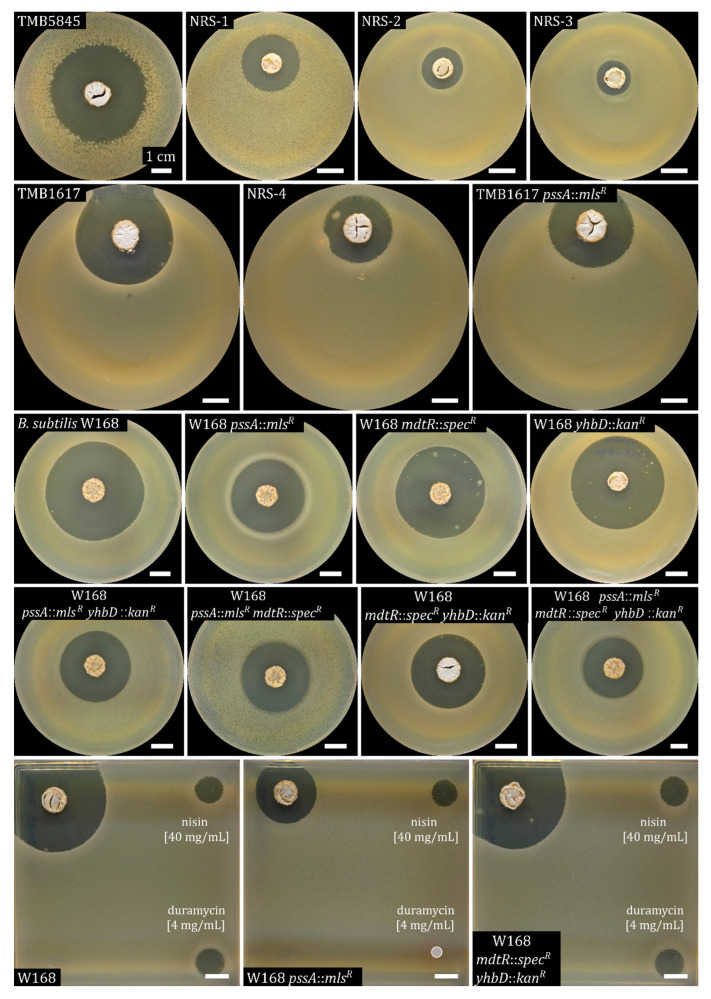
Antimicrobial activity against *B. subtilis* mutants. Inhibition was documented photographically using a P.CAM360 (1.48× magnification), and inhibition was measured. Scale bars indicate 1 cm. Duramycin [4 mg/mL] and nisin [40 mg/mL] were used as controls.

To evaluate the influence of mutations in these genes (*pssA*, *mdtR*, and *yhbD*) on the development of resistance, *B. subtilis* W168 mutants were constructed. The effects of deleting these genes individually and in different combinations on resistance to the antimicrobial compounds produced by the *A. coerulea* TMS085 were also investigated using the overlay method (Figure A7). The *pssA* deletion had the strongest impact on resistance, with the ZOI being reduced by approximately 50% (ZOI approx. 1.1 cm) in comparison to the *B. subtilis* W168. Deletion of transcriptional repressors (*mdtR*) for multidrug resistance genes reduced the ZOI to 1.6 cm (Figure 3C,D). The decrease in size of the inhibition zone after *yhbD* deletion, however, was measured as 0.2 cm in comparison to the *B. subtilis* W168 (Figure 3C,D). In contrast, the *yhbD* mutation is not solely responsible for the natural resistance of the strain NRS-3. The combined effect of the three mutations was also examined (Figure 3C,D). The results demonstrate that the resistance in the *pssA* mutant could be enhanced by the deletion of both *mdtR* (ZOI approx. 0.9 cm) and *yhbD* (ZOI approx. 1.0 cm). Although the double deletion of *yhbD* and *mdtR* influenced the development of resistance more strongly than the respective single-gene mutants, the combined effect of these mutations was less pronounced than that of the single *pssA* deletion (ZOI ∆*pssA* approx. 1.1 cm; ZOI ∆*mdtR* ∆*yhbD* approx. 1.2 cm). A triple-gene deletion displayed the strongest resistance to antibiotic substances produced by *A. coerulea* (ZOI approx. 0.8 cm).

### 2.4. Characterization of the Gene Cluster for Coerumycin, a Novel Cinnamycin Analog

**Figure A8:**
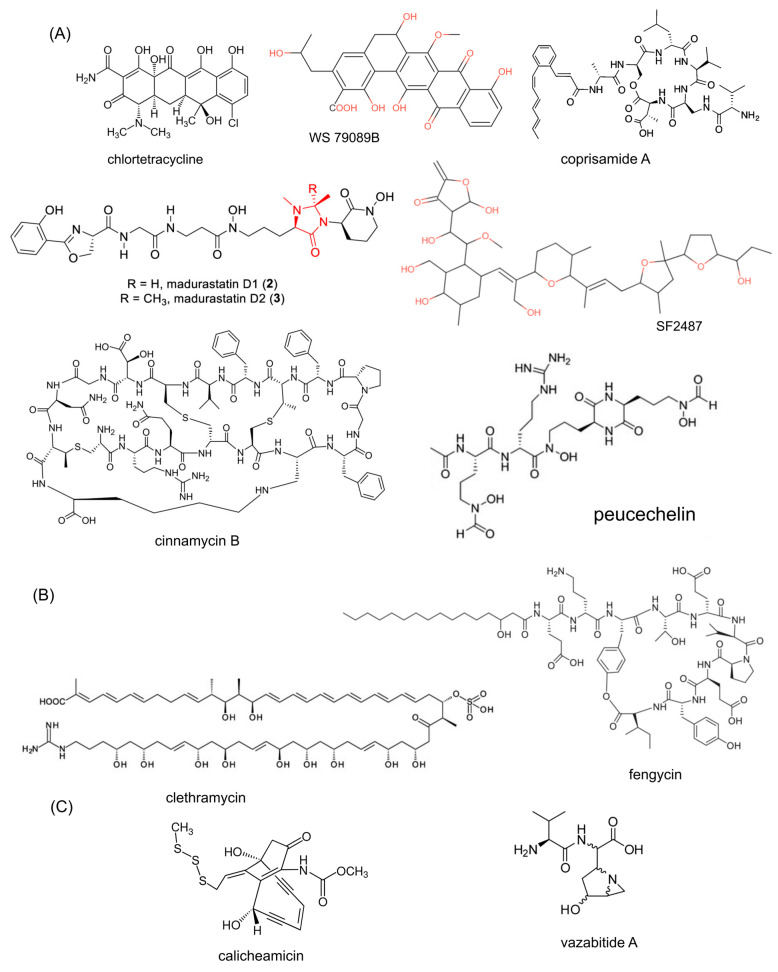
Structural formula of the potential secondary metabolites produced by *A. coerulea* TMS085, according to AntiSMASH analysis. (**A**) Compounds with antimicrobial activity (chlortetracycline (BGC0000209); WS 79089B (BGC0002732); madurastatin D1/madurastatin D2 [42]; SF2487 (BGC0002124); coprisamide C (BGC0002378); cinnamycin B (BGC0000503); peucechelin (BGC0002466)). (**B**) Compounds with antifungal activity (fengycin (BGC0001095); desulfoclethramycin (BGC0002498)). (**C**) Compounds with anticancer activity (calicheamicin (BGC0000033); vazabitide A (BGC0001818)).

For further characterization of the resistance developmental mechanism and identification of the produced lanthipeptides, an antiSMASH analysis was conducted on the assembled genome to identify the antimicrobial BGCs present in the strain TMS085 (Table A2, Figure A8). 23 BGCs were identified, including clusters for compounds with known antibacterial and antifungal activity that may be responsible for the synthesis of antibiotic substances with activity against Gram-positive bacteria (e.g., region 6 for the synthesis of madurastatins [42], region 12 and region 18 for coprisamide-like and cinnamycin-like lanthipeptides, respectively [43,44], and region 23 for Ni-siderophore-like peucechelin [45]). Also of note is the presence of BGCs for calicheamicin- and vazabitide-like anticancer agents (regions 7 and 15). Additionally, unidentified RIPP-like (region 2) and beta-lactone (region 9) clusters bearing no similarity to known BGCs were identified in the genome of the isolated strain. The low similarity between these identified gene clusters and known antimicrobial compounds suggests that the isolated *Actinomadura* species may be a potential source of new antibiotic substances.

**Figure 4:**
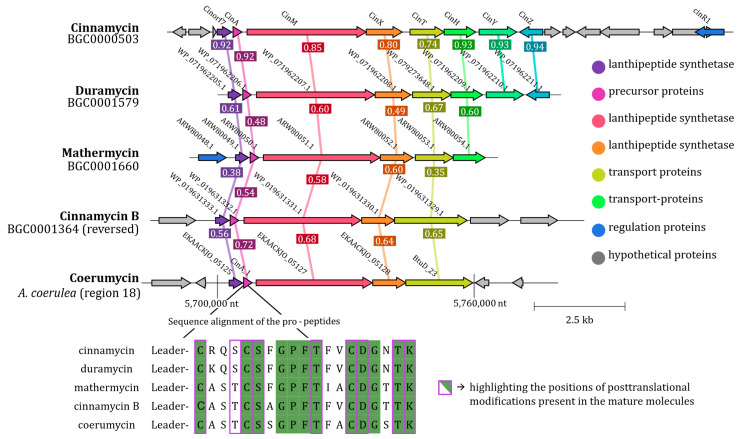
Comparative analysis of region 18 (BGC for coerumycin) as the novel cinnamycin-like lanthipeptide. The analysis was performed to the BGCs for cinnamycin (BGC00005203), duramycin (GDC0001579), mathermycin (BGC0001660), and cinnamycin B (BGC0001364). The identity between each gene is shown as [%/100%]. For the analysis of the lanthipeptide sequence, an alignment of the amino acid sequence of the CinA protein and its homologs was performed. The conserved amino acids are marked in green. The purple border marks the amino acids that are important for post-translational modifications and maturation of active antibiotics.

Considering the major influence of *pssA* mutations on the resistance of *B. subtilis* to antibiotics of the strain TMS085, the previously shown role of the *pssA* mutation in the duramycin resistance [46], and the established role of PssA in the synthesis of phosphatidylethanolamine (PE) as a natural component of the bacterial plasma membrane and specificity target for the cinnamycin-like lanthipeptides (e.g., duramycin), focus was laid on the identification of the BGCs for the cinnamycin-like lanthipeptides in the genome of *A. coerulea*. AntiSMASH identified a gene cluster (region 18) with 26% similarity to the BGC for cinnamycin (see Supplementary Materials Dataset S4). The identified BGC was aligned with four known BGCs for molecules structurally related to duramycin (cinnamycin, BGC00005203; duramycin, GDC0001579; mathermycin, BGC0001660; cinnamycin B, BGC0001364; Figure 4) [47].

The identified gene cluster is composed of five genes, including two biosynthetic core genes (EKAACKJO_05127 and EKAACKJO_0528) that play a pivotal role in the post-translational modification of pre-pro-lanthipeptides. These two genes exhibit the highest similarity to their homologs in the reference gene clusters, with respective percentage identities of 68% and 64%. The high degree of similarity between the BGCs for cinnamycin-like lanthipeptide was additionally confirmed by gene synteny. The BGC of strain TMS085 also contains a gene (*bluD*_23) with 65% similarity to the reference gene for transport proteins in the cinnamycin B gene cluster (Figure 4). Interestingly, in three additional BGCs, two genes were identified as responsible for the transport system for synthesized lantibiotics. Through protein alignment, it was confirmed that the predicted gene contains the sequences for both transport proteins from the cinnamycin BGC. A gene for a hypothetical protein (EKAACKJO_05125) was identified in all gene clusters. These three key biosynthesis genes (EKAACKLO_05125, EKAACKLO_05127, and EKAACKLO_05128) were identified as homologs of Cinorf7, CinM, and CinX, respectively. These genes are involved in critical post-translational modification steps (e.g., formation of lysine-alanine bridge between Lys19 (K19) and dehydroalanine (dehydrated S6), dehydrogenation of serine (S6) and threonine (T4, T11, and T18), and hydroxylation of Asp15 (D15)) in the synthesis of lanthipeptides. Lastly, a gene for a precursor peptide (*cinA*_1) was identified within the genome of the isolated strain. This prepropeptide determines the sequence of the cinnamycin analog. For this, the amino acid sequences of the leader peptide and the pre-pro-peptide were identified. The leader peptide sequence is 63 amino acids long from the N-terminus and is cleaved off during modification at the conserved amino acid motif shown by underlines in Figure 4. The structural pro-peptide sequences were conserved, and the sequence of -CxxxCSxGPFTxxCDGxTK was found to be a common motif for cinnamycin analogs (Figure 4). While the majority of amino acids involved in the posttranslational modifications of this lanthipeptide in the novel analog are identical to those observed in the other reference peptides, notable discrepancies are evident in the amino acids involved in the synthesis of the novel cinnamycin analog, which was therefore named coerumycin.

### 2.5. pssA-Mutations and Subsequent Resistance Development Against Cinnamycin Analogs

**Figure A9:**
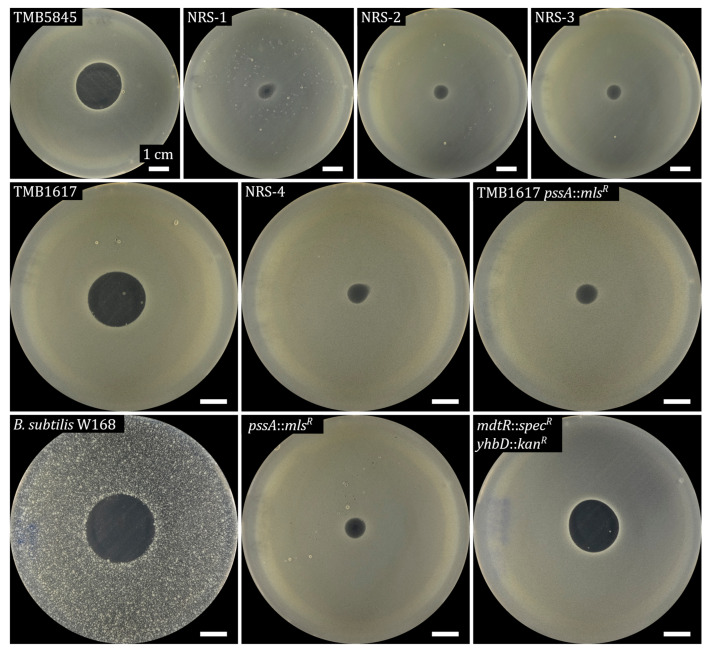
Antimicrobial activity of duramycin [2 mg/mL] against *B. subtilis* mutants. Inhibition was documented photographically using a P.CAM360 (1.48× magnification), and inhibition was measured. Scale bars indicate 1 cm.

Next, we analyzed the impact of the *pssA* mutation on the emergence of resistance to cinnamycin analogs. Both duramycin and supernatant from an eleven day old culture of the *A. coerulea* strain TMS085 inhibited the growth of *B. subtilis* W168 and TMB5845 (Figure A7), while both spontaneous mutant and *pssA* deletion strains displayed resistance to duramycin. It was additionally demonstrated that the deletions of *mdtR* and *yhbD* had no impact on the resistance of the bacterial strain to the duramycin and supernatants after cultivation of *A. coerulea* TMS085 (Figure A7 and Figure A9).

**Figure 5:**
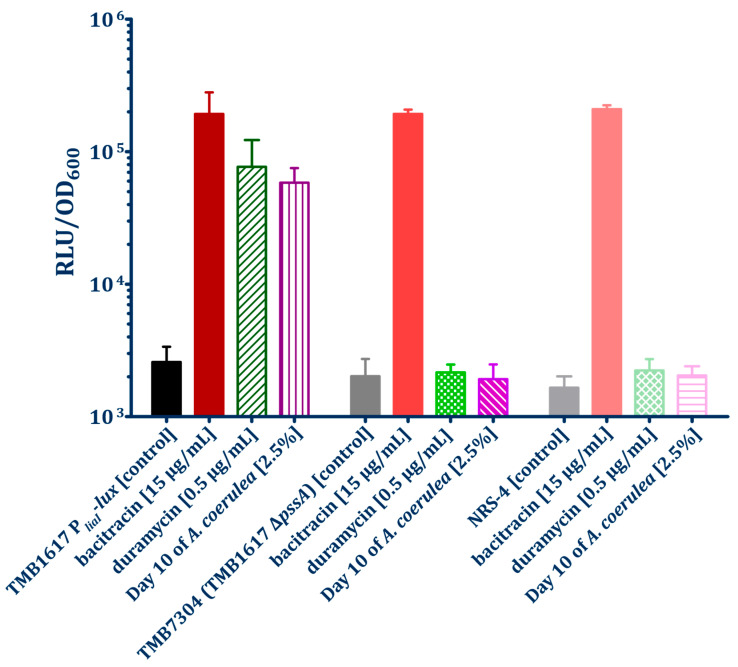
Quantification of biosensor promoter induction in *pssA*-mutants TMB7304 and NRS-4 by the isolate supernatants (Day 10) was performed in plate reader assays measuring bacterial growth (as optical density at 600 nm (OD_600_)) and luminescence of the biosensors. Induction via the addition of supernatants or controls occurred at time point 60 min. Average relative luminescence units (RLU) and their standard deviations are depicted for TMB1617 to the time point 30 min post-induction (90 min). Controls consisted of the uninduced biosensor with the addition of 5 µL sterile LB. Bacitracin and duramycin (in the final concentration per well 15 µg/mL and 0.5 µg/mL; as the concentrations that showed no negative effects on the growth of the biosensors during the experiments (Figure A11)) were used as the positive control. Plate reader assays were performed in biological and technical triplicates for each strain and cultivation time point.

**Figure A10:**
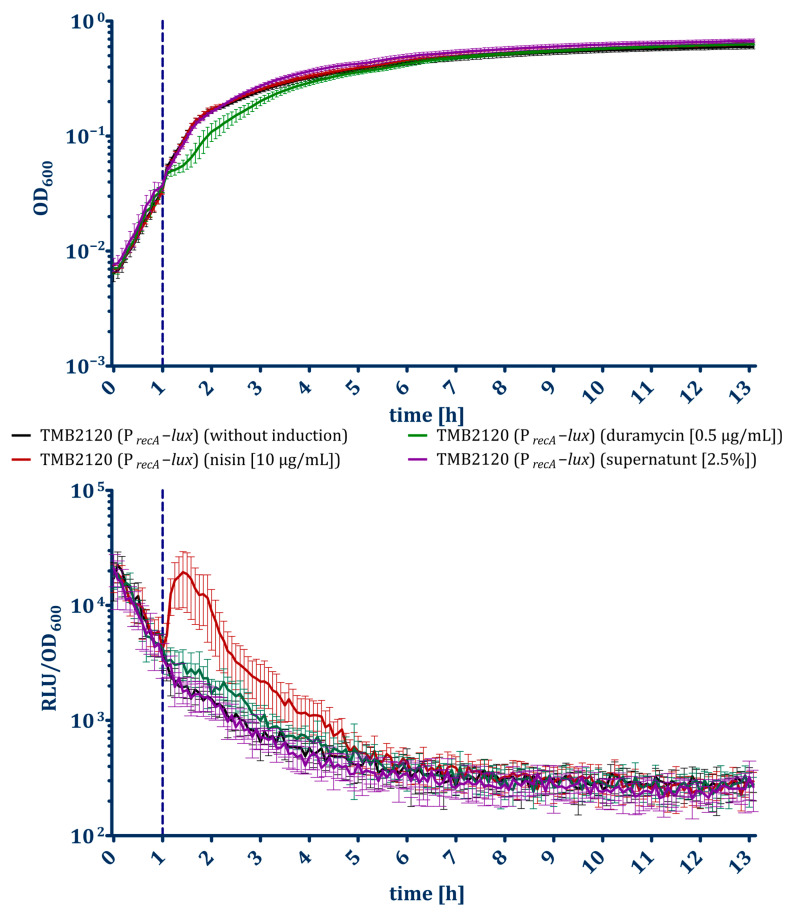
Quantification of biosensor promoter induction in TMB2120 by the isolate supernatants (Day 10) and duramycin [0.5 µg/mL] was performed in plate reader assays measuring bacterial growth (as optical density at 600 nm (OD_600_)) and luminescence of the biosensors. Induction via the addition of supernatants or controls occurred at time point 60 min (dashed blue line). Controls consisted of the uninduced biosensor with the addition of 5 µL sterile LB. Nisin (in the final concentration per well of 10 µg/mL) was used as the positive control. Plate reader assays were performed in biological and technical triplicates for each strain and cultivation time point.

**Figure A11:**
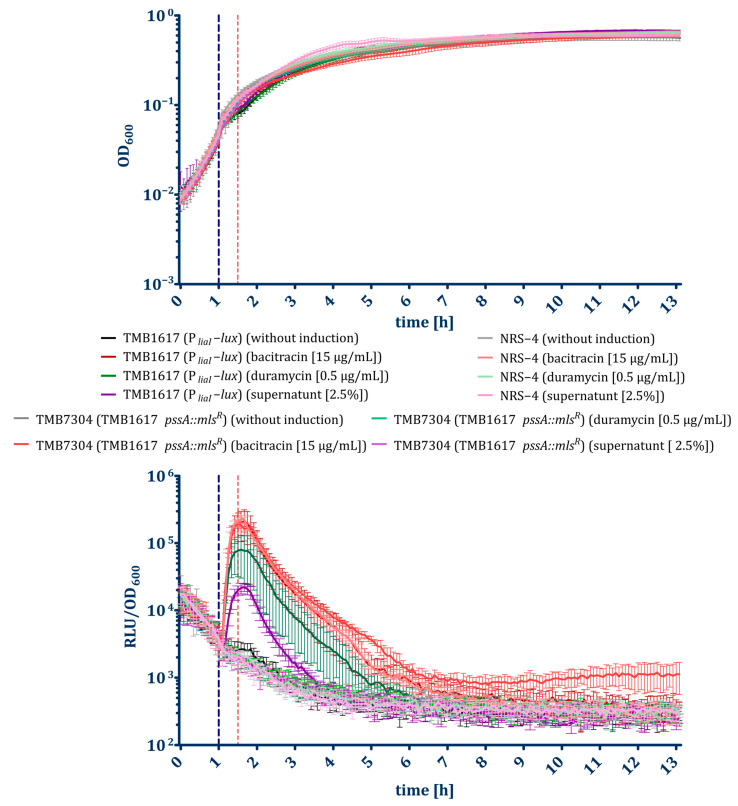
Quantification of biosensor promoter induction in TMB1617 and *pssA*-mutants TMB7304 (TMB1617 *pssA*::*mls^R^*) and NRS-4 by the isolate supernatants (Day 10) was performed in plate reader assays measuring bacterial growth (as optical density at 600 nm (OD_600_)) and luminescence of the biosensors. Induction via the addition of supernatants or controls occurred at time point 60 min (dashed blue line). Average relative luminescence units (RLU) and their standard deviations are depicted for TMB1617 to the time point 30 min post-induction (90 min, dashed red line). Controls consisted of the uninduced biosensor with the addition of 5 µL sterile LB. Bacitracin and duramycin (in the final concentration per well 15 µg/mL and 0.5 µg/mL) were used as the positive control. Plate reader assays were performed in biological and technical triplicates for each strain and cultivation time point.

The antibacterial effect of the cinnamycin analogs was characterized using the biosensors TMB1617 and TMB2120, which were induced by the secondary metabolites of *A. coerulea* TMS085. The studies demonstrated that the addition of a sub-inhibitory concentration of duramycin [0.5 µg/mL] did not induce the P*_psdA_*-*luxABCDE* biosensor TMB2120 (Figure A10), in accordance with the preceding biosensor assay utilizing sterile supernatants from cultivated *A. coerulea*. In contrast, the induction of the P*_liaI_*-*luxABCDE* biosensor TMB1617 by duramycin was observed, which also correlates with the induction of this biosensor by sterile supernatants (Figure 5 and Figure A11). Following the addition of duramycin, an approximately 29-fold increase in the normalized RLU values compared to the uninduced biosensor was detected after 30 min. Furthermore, biosensor assays were performed with the TMB1617 *pssA* mutants (NRS-4 and TMB7304), whereby both strains showed no induction by the addition of duramycin and sterile supernatant of cultured *A. coerulea* (Figure 5 and Figure A11).

## 3. Discussion

The discovery of new or endemic actinomycetes from previously unexplored ecological niches is a promising strategy for the isolation of novel antimicrobial compounds. Although most commonly associated with soil, recent research has focused on associations between antimicrobial-producing actinomycetes and animals [13,14,15]. In this work, an antibiotic-producing actinobacterium was isolated from meerkat fecal samples (*Suricata suricatta*). These small mongooses live primarily in arid regions of southern Africa, where the diurnal animals spend much of their time in the sand. Their natural diet consists primarily of insects, arachnids, and small vertebrates. Both their terrestrial lifestyle and natural diet make examining fecal samples from these animals interesting regarding the isolation of possible antibiotic-producing bacteria. The isolate displayed macroscopic and microscopic characteristics typical of actinomycetes, including filamentous growth and differentiation of colonies into complex substrate and sporulating aerial mycelium structures [5].

Through whole-genome sequencing and subsequent phylogenetic analysis, the isolate was identified as *A. coerulea*, with multilocus sequence analysis (MLSA) evolutionary distances (ED) of 0.008 between the natural isolate and other *A. coerulea* reference strains (DSM 43675 and JCM 3320) [48]. However, subsequent analysis of the 8.2 Mbp genome showed significant differences in comparison to that of the reference strain, indicating a higher degree of genetic variability within this species. The genomes differ in length (8,188,457 bp for the isolate vs. 8,190,213 bp for the reference) and GC content (72.55% vs. 72.59%) as well as the number of CDSs (7908 vs. 7883 CDSs), with most of the additional genes in the isolated strain having a hypothetical function. A total of 141,833 nucleotide variations were identified between the two genomes. This high genetic variability correlates with the lower similarity found between the proteomes of the two strains, as only 45.6% of all *A. coerulea* proteins showed >98% similarity to the proteins of the reference strain. Considering the above findings of high genomic and phylogenetic similarity combined with increased genetic and protein variation, it can be assumed that the strain is a new *A. coerulea* strain.

*Actinomadura* species belonging to the genera *Actinomycetota* are distinguished not only by their filamentous growth, sporulation, and lack of motility, but also by their capacity to produce multiple secondary metabolites with a broad range of activities [20]. Despite its initial isolation in 1975 by Preobrazhenskaya et al., *A. coerulea* remains a relatively understudied species, particularly in terms of the antimicrobial compounds it produces [48]. Subsequent genome analysis of *A. coerulea* TMS085 identified 23 different secondary metabolite biosynthesis gene clusters (smBGCs), most of which showing low similarity (<60%) to known antibiotic gene clusters (Table A2 and Figure A8).

The isolated *A. coerulea* strain displayed moderate activity against pathogenic yeasts (e.g., *C. neoformans* and *E. dermatitidis*) and filamentous fungi (e.g., *P. chrysogenum* and *A. niger*), and two BGCs (regions 4 and 10) with similarity to two known gene clusters for antifungal compounds (fengycin and clethramycin) were identified in the genome of strain TMS085. Subsequent in-depth analysis, however, failed to confirm any similarity in the core biosynthesis genes between the isolate and the reference clusters. Gene clusters homologous to the unknown betalactone antibiotic were, however, identified in other *Actinomadura* species, including *Actinomadura madurae* MRC008, *Actinomadura glauciflava* DSM 44770, and *Actinomadura oligospora* ATCC 43269 P696D. An entirely novel gene cluster for a betalactone (region 9) was discovered in the genome of *A. coerulea* TMS085, and its homologs were also identified in other *Actinomadura* species (e.g., *Actinomadura glauciflava*). In all identified gene clusters, the potential transcriptional binding sites for secondary metabolite regulators such as DasR (N-acetylglucosamine-dependent repressor) and OsdR (developmental and stress regulator) were also detected, indicating that these gene clusters are activated under stress conditions (e.g., cell-cell-interaction with other microorganisms).

The secondary metabolites produced by the *A. coerulea* TMS085 demonstrated notable antibacterial activity against Gram-positive species, including *B. subtilis*, *B. mycoides*, and *S. aureus*. Genome mining identified the presence of 12 different BGCs for antibacterial compounds (e.g., polyketide synthase (PKS)- and nonribosomal peptide synthetases (NRPS)-BGCs as well as genes for the synthesis of siderophores and lanthipeptides). While the majority of these BGCs exhibit only low to intermediate similarity to reference clusters for antibiotics with activity against Gram-positive bacteria, it should be noted that not all of these gene clusters are likely expressed under laboratory conditions. This phenomenon can be attributed to the distinctive characteristics of the *Actinomycetales*, which have been observed to activate specific smBGC gene clusters in response to specific growth and stress conditions [5]. To identify and characterize naturally produced antibiotic compounds, the whole-cell biosensor platform used in this work can be utilized. Antibiotic-specific activation of specific promoters in bacterial cells, such as in *B. subtilis*, further facilitate the identification of the cellular targets of antimicrobial compounds, thereby providing insights into potential modes of action. Based on the specific induction of the biosensors, the spectrum of naturally produced compounds after seven days of incubation could be narrowed down and the resulting activation of secondary metabolism in the naturally produced lanthipeptides and other cell wall antibiotics (Figure 2, Figure A4, Figure A5 and Figure A6).

The strong selective pressure of antibiotic compounds often results in spontaneous mutations that facilitate the resulting strain resistance, thereby conferring a selective advantage. The nature of such mutations could also provide information on the nature of the antibiotics that have provoked them. For these reasons, the characterization of the natural evolution of resistance represents another powerful strategy for understanding resistance mechanisms as well as identifying and characterizing naturally produced antibiotic metabolites [49,50]. The direct selection of *B. subtilis* for increased resistance to naturally produced antibiotic compounds of *A. coerulea* was therefore conducted. Using genome analysis and subsequent construction of mutants, non-silent mutations in three genes (*pssA*, *mdtR*, and *yhbD*) were identified as resistance-promoting mutations. By constructing single-gene deletion strains, the influence of the individual mutations on the development of resistance could be characterized. The resulting increased resistance of *B. subtilis* multi-gene deletion strains confirmed the combined effects of these mutations.

Mutation and deletion of *yhbD* demonstrated a minimally positive effect on the resistance to antibiotic compounds of *A. coerulea*. Although *yhbD* is part of the *yhbDEF* operon for bactofilin synthesis, the precise function of this gene is still unknown. Bactofilins are a largely conserved protein family involved in the formation of flagellar hooks and flagellar filaments in *B. subtilis* [51]. The position of *yhbD* suggests that this protein may have a potential regulatory function in the expression of the operon. Its potential role of this natural nonsense mutation in the development of resistance remains to be unraveled.

The *mdtR* mutants also showed increased resistance to *A. coerulea* antibiotics in comparison to the wild-type strain. In *B. subtilis*, MdtR is a natural repressor of the expression of the gene for multidrug efflux transporter (*mdtP*). This protein contributes to resistance to several antibiotics, including fusidic acid, novobiocin, streptomycin, and actinomycin, possibly by pumping these structurally unrelated antibiotics out of the cells [52]. The expression of *mdtP* is inhibited by the DNA binding of *mdtR*, which possesses a helix-turn-helix motif. The deletion of five nucleotides in the native sequence, the alteration of the protein sequence within the helix-turn-helix motif of *mdtR*, and the deletion of *mdtR* itself were observed to increase *mdtP* expression. This, in turn, was found to enhance the natural non-specific antibiotic resistance observed in the *B. subtilis* strains. However, the functionality of this mutation is limited by the concentration of antibiotics produced, which also explains the only moderate effect of the singular *mdtR* mutation on resistance.

In response to the presence of antibiotics, the expression of specific genes initiates defensive mechanisms crucial for ensuring survival [53,54,55,56]. As one example, the so-called cell envelope stress responses are induced in the presence of cell wall-targeting antibiotics (e.g., nisin and bacitracin) [57]. The bacterial cell envelope is a fundamental, multi-layered, and intricate structure that physically separates bacteria from their surrounding environment and serves as the first potential target for antimicrobial compounds. The cell membrane represents the functional barrier of the cell and is critical for the generation of a proton motive force, thereby providing an interface for protein-protein interactions and acting as a diffusion barrier [58]. Given the physiological importance of the bacterial cell membrane, monitoring its integrity and, if necessary, mounting appropriate responses against cell membrane-damaging compounds are of critical importance for maintaining its functionality [55]. Our data demonstrated that the primary resistance detected against *A. coerulea* was associated with *pssA*. Phosphatidylserine synthase (PssA) is an important membrane-bound enzyme for the synthesis of phosphatidylethanolamine (PE), a natural component of the bacterial plasma membrane [59]. This enzyme catalyzes the synthesis of phosphatidylserine, the natural precursor of PE, by modifying CDP-diacylglycerol molecules with serine [46]. The natural *pssA* mutation leads to a change in an amino acid, which can negatively affect PE synthesis. Accordingly, no PE could be detected in the plasma membrane of this mutant. PE molecules represent a specific target for certain lanthipeptides [60,61,62,63], e.g., cinnamycin-like lanthipeptides, which have been reported to specifically form an equimolecular complex with PE. The binding of cinnamycin to PE results in the depolarization of the plasma membrane by pore formation. Given that the deletion of *pssA* also halts the synthesis of PE molecules and that strains with diminished PE content exhibit heightened resistance to cinnamycin-like lanthipeptides, it was hypothesized that the increased resistance of the *pssA* mutants functions specifically in terms of the cinnamycin-like lanthipeptides produced by *A. coerulea*. Testing of duramycin, a lanthipeptide antibiotic structurally related to cinnamycin, confirmed the increased resistance of the natural *pssA* mutants of *B. subtilis*.

Subsequent experiments with duramycin and the sterile supernatants of *A. coerulea* demonstrated that these lantibiotics induced promoter activity and, thus, bioluminescence in the P*_liaI_*-based biosensor TMB1617 for cell envelope stress (Figure 3, Figure 5, Figure A5 and Figure A11). This biosensor contains the *lux* cassette fused to the *liaI* promoter, which is controlled by the LiaRS system in *B. subtilis*, which plays a pivotal role in the cellular response to cell wall and membrane stress [64]. It has been previously demonstrated that antibiotics that disrupt the lipid II cycle, including bacitracin, nisin, and daptomycin, induce the Lia response in *B. subtilis* [65,66]. Primary resistance to bacitracin was conferred by TCS-BceRS-dependent activation of the ABC transporter BceAB [67,68]. In contrast, the cinnamycin-like antibiotics bind to PE lipids, thereby disrupting the overall fluidity of the membrane in a manner analogous to daptomycin [66]. This, in turn, can lead not only to membrane depolarization but also to delocalization of membrane-bound proteins involved in lipid II synthesis (e.g., MurG), ultimately resulting in disruption of cell wall synthesis. Based on the natural role of *liaIHGFSR* as a cell envelope stress response system, such indirect-acting antibiotics could also lead to induction of the P*_liaI_*-*lux* biosensor [66]. Most importantly, introducing a *pssA* deletions or the spontaneous loss-of-function mutation of this gene in the P*_liaI_*-*lux* biosensor result in a substrate-specific loss of bioluminescence, due to a loss of the cellular target of duramycin and a related compound found in *A. coerulea* supernatants (Figure 5 and Figure A11). This specific loss of bacterial stress readout, without affecting the functionality of the biosensor, as demonstrated by the unchanged induction of the P*_liaI_*-*lux* biosensor and hence bioluminescence after the addition of bacitracin underscores the role of PssA in the development of resistance and provides a strong indication of the natural production of a cinnamycin-like lanthipetide in *A. coerulea* TMS085.

Subsequently, genome mining identified a gene cluster with 26% similarity to the known gene clusters of cinnamycin and its analogs. Comparative analysis of the pre-pro-lanthipeptide genes confirmed that the identified region 18 is responsible for the synthesis of a new cinnamycin analog designated “coerumycin”. All cinnamycin-like BGCs (*cin*) contain five essential genes for lantipetide synthesis, including *cinA*, *cinM*, *cinX*, and *cinorf7*, as well as a substantial number of hypothetical proteins. The essential genes for lanthipeptide synthesis were also identified in the genome of *A. coerulea*, with CinA_1 coding for a pre-pro-coerumycin homologous to CinA. An -AYA- motif was identified in the aforementioned gene, which exhibited high similarity to the -AFA- motif present in CinA. This motif was also identified in the cinnamycin B gene cluster. It is postulated that it is recognized by the type I signal peptidase of the general secretion pathway, explaining the absence of a cinnamycin-specific protease in the cluster [69]. Three core biosynthesis genes (EKAACKJO_05125, EKAACKJO_05127, and EKAACKJO_05128) were also identified as homologs of Cinorf7, CinM, and CinX, respectively. The Cinorf7 homolog (EKAACKJO_05125) and all other Cinorf7 homologs form a lysine-alanine bridge between Lys19 (K19) and dehydroalanine (dehydrated S6). The CinM homolog (EKAACKJO_05127), which has a LANC-like dome and belongs to the lanthionine synthetase M family, catalyzes the dehydrogenation of serine (S6) and threonine (T4, T11, and T18) in pro-peptides, thereby forming lanthionine bridges [44,69]. EKAACKJO_05128 displayed a high degree of homology to CinX, the protein responsible for the hydroxylation of Asp15 (D15) on the precursor peptide. Following post-translational modification, coerumycin is most likely released from the hyphae by BtuD_23, an ABC transporter with ABC transporter and transmembrane domains, which exhibits homology to the CinTH protein. While the individual genes of the coerumycin-BGC (region 18) identified in this work exhibit a high degree of similarity (56–72%) to the genes responsible for the synthesis of cinnamycin analogs in other rare actinomycetes, a notable discrepancy was observed in the sequence of CinA_1 when compared to pre-pro-coerumycin. The amino acid sequence of coerumycin exhibited a greater diversity of amino acids outside the conserved motif for cinnamycin derivatives.

Despite the identification of all the genes involved in the synthesis and post-translational modification of cinnamycin homologues in the coerumycin gene cluster, the question of how coerumycin biosynthesis is regulated remains unanswered. O’Rourke et al. have demonstrated that cinnamycin biosynthesis is subject to complex and unique regulation [70]. Here, nutrient limitation leads to the production of smaller amounts of cinnamycin, which are exported from the cell by CinTH. This cinnamycin then activates the membrane-bound sensor kinase CinK, leading to the phosphorylation of CinR and the activation of *cinorf10* transcription. This monomethyltransferase enables autoimmunity by methylating the PE lipids in the membrane of *S. cinnamoneus*, thereby impeding the binding of the produced cinnamycin to the membrane. Production of active cinnamycin depends heavily on the degree of PE methylation, suggesting the presence of a post-transcriptional regulatory feedback mechanism. However, this raises additional questions about the regulatory pathways and autoimmunity involved in coerumycin production in *A. coerulea*. Furthermore, the further identification and purification of coerumycin from cultured *A. coerulea* through high-performance liquid chromatography (HPLC) were also severely limited by its low production as a highly biologically active lanthipeptide. Further experiments on the heterologous (over)expression of this gene cluster in *E. coli*, a Gram-negative bacterium, also produced inconclusive results, possibly due to the intricate and multifaceted regulation of gene expression within this cluster, which is contingent on cell wall sensing, a process that remains to be fully elucidated. A further constraint in the research on these antibiotics is the cryptic nature of their gene clusters, which results in minimal expression under standard laboratory conditions. However, this lower production is often compensated for by their very high biological activity (the minimum inhibitory concentration of cinnamycin and duramycin against *B. subtilis*, for example, is approximately 2 µM) [71,72]. The cryptic production, the not fully researched regulation, and the very high antibacterial activity of these lanthipeptides limit the classic isolation and structural characterization of such novel cinnamycin analogues as coerumycin, especially in connection with such rare actinomycetes as *A. coerulea* TMS085. However, coerumycin and other cinnamycin analogues remain promising candidates for further research due to their very high antibacterial and potential anticancer activity.

Taken together, it is tempting to suggest that the natural metabolites produced by endemic *Actinomycetales* species continue to represent a promising source for the discovery of compounds with high structural diversity and different bioactivities. Further research into rare actinomycetes could ultimately lead to significant advances in microbiology and biotechnology, opening up new avenues for combating infectious diseases and addressing the antibiotic crisis.

## 4. Materials and Methods

Oligonucleotides used in this study are shown in Table 2.

### 4.1. General Growth Conditions

All wild-type/laboratory and whole-cell biosensor strains were cultivated in lysogeny broth (LB-Medium (Luria/Miller), Carl Roth GmbH + Co., KG, Karlsruhe, Germany) under aerobic conditions at 37 °C with agitation (180 rpm) unless specified otherwise. For solid agar plates, 1.5% (*w*/*v*) (0.75% for soft agar) agar-agar was added. *B. subtilis* cells carrying a resistance marker were selected for using chloramphenicol (5 μg/mL), kanamycin (10 μg/mL), spectinomycin (100 μg/mL), and/or erythromycin combined with lincomycin (1 μg/mL, 25 μg/mL) for MLS. Transformation of *B. subtilis* was performed as previously described [73]. The yeasts and fungi used in this work were cultivated under aerobic conditions at 30 °C with shaking (180 rpm) unless specified otherwise. The fungal strains (e.g., *Penicillium chrysogenum*, *Mucor racemosus*, *Rhizopus microspores*, *Aspergillus niger*, and *Scopulariopsis brevicaulis)* was kept as a spore suspension at −20 °C and used for the inoculation of soft agar (100 μL in 10 mL LB soft agar). The isolated *Actionomadura* strain was cultivated on GAU medium [74] or maltose-yeast extract-malt extract (MYM) medium prepared with tap and deionized water (1:1) and supplemented with 200 mL of R2 trace element solution (ZnCl_2_ (40 mg/L), FeCl_3_·6H_2_O (200 mg/L), CuCl_2_·2H_2_O (10 mg/L), MnCl_2_·4H_2_O (10 mg/L), Na_2_B_4_O_7_·10H_2_O (10 mg/L), (NH_4_)_6_Mo_7_O_24_·4H_2_O (10 mg/L)) per 100 mL medium [75]. Liquid cultures were incubated aerobically at 28 °C and 170 rpm. Colonies were spotted using 10 μL spore suspension and incubated under these conditions on MYM agar for seven days prior to all bacterial and fungal overlays.

### 4.2. Isolation and Morphological Characterization of Actinobacteria from Suricata suricatta Fecal Samples

The fecal samples utilized in this work were obtained by animal caretakers (Zoo Dresden, Dresden, Germany) following defecation and placed into sterile 50 mL centrifugation tubes that were refrigerated until processed. Subsequently, 1 g of each sample was resuspended in 10 mL sterile ultrapure water. 1 mL of this suspension was added to 9 mL of a 1.4% (*v*/*v*) phenol solution and incubated at room temperature for one hour. 100 μL of the sample were plated on GAU agar and incubated for seven days at 28 °C. Single colonies bearing morphology typical for actinobacteria (white, gray, or brown coloring, visible complex substrate and aerial mycelium structures) were picked and streaked onto MYM agar. Created spore suspensions were stored at −20 °C. Spore suspensions of isolated strains were spotted on MYM agar plates and incubated according to the general growth conditions. Pictures of colonies were taken on a black background using a P.CAM360 (1.48× magnification, overhead light level 3) [TUD, Dresden, Germany] and edited for light and contrast using the open-source software Fiji [76]. Liquid culture of the isolated *Actinomadura coerulea* was applied to 1% (*w*/*v*) “UltraPure agarose” (Invitrogen, Thermo Fisher, Waltham, MA, USA) pads and phase contrast microscopy was performed using an Axio Observer 7 inverse microscope (Carl Zeiss Microscopy GmbH, Jena, Germany).

### 4.3. Whole-Genome Sequencing and Analysis

Isolation of genomic DNA from the isolated strains was performed using the MACHEREY-NAGEL NucleoSpin^®^ Microbial DNA kit (Düren, Germany) with modifications to the standard kit protocol: 10 mL culture were inoculated with spore suspension of isolated strain to the final concentration of 10^6^ spores/mL and incubated under standard conditions. Cell pellets were concentrated by repeated rounds of centrifugation and decanting of the supernatant. 50 μL lysozyme (15 mg/mL, Sigma-Aldrich, St. Louis, MO, USA) were added to the cell pellets during the lysis step and samples were shaken in a mixer mill (Retsch, Haan, Germany) for 20 min at 4 °C. Elution of genomic DNA was performed with 50 μL of the provided elution buffer. Strain was sequenced by the sequencing service of the LMU Biozentrum (Martinsried, Germany) using Illumina 1.9 paired-end and Oxford Nanopore sequencing methods.

Genome alignment and analysis for the isolated *A. coerulea* strain was performed using the open-source platform Galaxy EU (24.1). The quality of the Illumina paired-end data was verified with FastQC (Galaxy v.0.11.8) [77]. To automate quality and adapter trimming, the sequencing data were analyzed with Trim Galore! (Galaxy v.0.4.3.1; threshold: 20; maximum error rate: 0.1; minimum reads length: 20) and sequences bearing an average quality below the threshold value were subsequently removed using Trimomatic (Galaxy v.0.36.5; Average quality required: 20; Number of bases to average across: 4) [78]. High-quality paired-end data were then assembled as contigs using Unicycler (Galaxy v.0.4.8.0; minimum length of contigs: 100 bp) [79]. The assembled genome was identified using the web-based Type (Strain) Genome Server (TYGS; https://tygs.dsmz.de, accessed on 7 October 2024) using recently introduced methodological updates and features [30,80]. Tree inference was carried out according to the Genome BLAST Distance Phylogeny Approach with branch support values from pairwise whole-genome or single-gene distances and calculated dDDH values used for species and subspecies delineation [30,81]. The whole-genome-based phylogenetic tree was created using iTOL [33]. Subsequently, the genome assembly contigs were aligned with the most closely related genome (*A. coerulea* DSM 4367; RefSeq: GCF_014208105.1) using the RagTag tool (reference-guided scaffolding of draft genomes; Galaxy Version 2.1.0 + galaxy1) [79,82]. Information on nomenclature, synonymy, and associated taxonomic literature was provided by TYGS’s database [80]. The results were provided by the TYGS on 7 November 2024. The TYGS analysis was subdivided into the following steps: determination of closely related type strains, pairwise comparison of genome sequences, phylogenetic inference, and type-based species and subspecies clustering.

Determination of closest type strain genomes was done in two complementary ways: Firstly, all user genomes were compared against all type strain genomes available in the TYGS database via the MASH algorithm, a fast approximation of intergenomic relatedness, and the ten type strains with the smallest MASH distances were chosen per user genome [83]. Secondly, an additional set of ten closely related type strains was determined via the 16S rDNA gene sequences. These were extracted from the user genomes using RNAmmer [84] and each sequence was subsequently BLASTed [85] against the 16S rDNA gene sequence of each of the 21,339 type strains currently available in the TYGS database. This was used as a proxy to find the best 50 matching type strains (according to the bitscore) for each user genome and to subsequently calculate precise distances using the Genome BLAST Distance Phylogeny approach (GBDP) under the algorithm ’coverage’ and distance formula d5 [86]. These distances were finally used to determine the 10 closest type strain genomes for each of the user genomes. For the phylogenomic inference, all pairwise comparisons among the set of genomes were conducted using GBDP and accurate intergenomic distances inferred under the algorithm ’trimming’ and distance formula d5 [86]. 100 distance replicates were calculated each. Digital DDH values and confidence intervals were calculated using the recommended settings of the GGDC 4.0 [80,86]. The resulting intergenomic distances were used to infer a balanced minimum evolution tree with branch support via FASTME 2.1.6.1 including SPR postprocessing [32]. Branch support was inferred from 100 pseudo-bootstrap replicates each. The trees were rooted at the midpoint and visualized with PhyD3 [87,88]. The type-based species clustering using a 70% dDDH radius around each of the 16 type strains was done as previously described [80]. Subspecies clustering was done using a 79% dDDH threshold as previously introduced [81].

### 4.4. Analysis of Assembled A. coerulea Genome

The assembled genome of the isolated *A. coerulea* strain was annotated with the “prokka” software tool (Galaxy Version 1.14.6 + galaxy1), BV-BRC genome annotation tool (v.3.37.14), and BV-BRC comprehensive genome analysis service at PATRIC (v.3.37.14) [34,89,90,91,92,93,94,95,96]. The predicted proteomes for the isolated *A. coerulea* and the reference strain DSM 4367 (RefSeq: GCF_014208105.1) were determined and compared using the BV-BRC proteome comparison tool under the standard parameters [34,97]. Comparison of orthologous gene clusters and their respective proteins was conducted using the OrthoMCL clustering algorithm under default parameters from OrthoVenn3 [98]. To determine genomic similarity and identify variations, the sequencing paired contigs of the isolated strain after Trimmomatic analysis were aligned to the genome of the reference strain DSM 4367 (RefSeq: GCF_014208105.1) using the snippy tool with the default parameters (Galaxy Version 3.2) [36]. Visualization of the assembled data and the localization of single-nucleotide polymorphisms (SNPs) and insertions/deletions was performed using JBrowse genome browser (Galaxy Version 1.16.11 + galaxy1) [99]. The genome of the isolated strain was screened using AntiSMASH (v. 7.0) under default parameters for the presence of known biosynthetic gene clusters for secondary metabolites [100]. Alignment and comparison analysis of identified gene clusters for the antimicrobial compounds were performed with clinker (https://cagecat.bioinformatics.nl/, accessed on 20 July 2024) [101] using standard input parameters and CLC Main Workbench 7 (Qiagen, Hilden, Germany).

### 4.5. Spot-on-Lawn and Overlay Assays

The antimicrobial activity of the produced compounds was tested by plate spreading soft agar inoculated with a bacterial or fungal strain onto MYM plates containing colonies of the isolated *A. coerulea* strain. An initial inhibition test was conducted with bacteria (Gram-positive *Bacillus subtilis* W168, *Bacillus mycoides* ATCC 6462, *Staphylococcus aureus* ATCC 25923 and Gram-negative *Escherichia coli* K12, *Pseudomonas aeruginosa* ATCC 27853, *Proteus mirabilis*) as well as the filamentous fungus (*Penicillium chrysogenum*, *Mucor racemosus*, *Rhizopus microspores*, *Aspergillus niger*, and *Scopulariopsis brevicaulis*) and pathogenic yeasts (*Cryptococcus neoformans* ATCC 90112, *Exophiala dermatitidis*, *Candida tropicalis*, and *Candida guilliermondii*). All experiments involving BSL-2 microorganisms (*B. mycoides* ATCC 6462, *S. aureus* ATCC 25923, *P. aeruginosa* ATCC 27853, *P. mirabilis*, *M. racemosus*, *R. microspores*, *S. brevicaulis*, *A. niger*, *C. neoformans* ATCC 90112, *E. dermatitidis*, *C. tropicalis*, and *C. guilliermondii*) were conducted in a BSL-2 laboratory (Institute for Microbiology, TU Dresden, Dresden, Germany). Potential antimicrobial substance classes of metabolites produced by the natural isolates were determined by measuring bioluminescence produced by a panel of eight whole-cell biosensors (Table 3). These modified *B. subtilis* strains include the genomically integrated luminescence reporter cassette *luxABCDE* under the control of an inducible antibiotic-specific promoter. Detection and intensity of bioluminescence were used to characterize antimicrobial compounds based on their cellular targets.

Overnight cultures were inoculated in LB with the corresponding antibiotic when required. Day cultures of the antagonist or biosensor strains were inoculated 1:250 in fresh LB w/o antibiotic from overnight cultures on the following day and grown to an OD_600_ of 0.4–0.9. These were subsequently used to inoculate 10 mL of melted LB soft (0.75%) agar to achieve a final OD_600_ of 0.01 and poured onto the surface of the MYM plates and around the pre-grown isolate colonies. The antimicrobial activity was tested by spotting 10 μL of either sterile filtered culture supernatants on top of the bacterial or fungal lawn [39,102]. Sterile filter discs soaked in 10 μL of either an antibiotic (positive control; nisin (40 mg/mL) for *B. subtilis* W168, ciprofloxacin (200 µg/mL) for all pathogenic bacterial strains, norfloxacin (100 µg/mL) for *E. coli* K12, amphotericin B (250 µg/mL) for the fungi and yeasts, and cycloheximide (1 mg/mL) for *C. neoformans*) or a 50% (*v*/*v*) glycerin solution (negative control colony overlays) or 95% (*v*/*v*) methanol (negative control fraction spot-on-lawn testing) were applied to the surface of the plates after spread coating. For the whole-cell biosensor assays, positive and negative controls consisted of sterile filter discs soaked in 10 μL of the corresponding antibiotics (Table 3) and a 50% (*v*/*v*) glycerin solution or 95% (*v*/*v*) methanol. Overlay plates were incubated overnight according to the standard conditions listed for bacteria and yeast/fungi. Plates were documented photographically on a black background using a P.CAM360 (1.48× magnification, overhead light level 3) [TU Dresden], and zones of inhibition (ZOI) were measured. The luminescence output of the biosensor strains was measured using the chemiluminescence imaging system Fusion FX from Vilber Lourmat Deutschland GmbH (Eberhardzell, Germany) with an exposure time of one minute. Images were cropped and adjusted for brightness and contrast using Fiji [76]. The role of loss-of-function mutations in natural resistance of *B. subtilis* was verified by plate-spreading soft agar inoculated with the mutant strains onto MYM plates containing *A. coerulea* colonies or via spot-on-lawn assays with supernatants of isolated *A. coerulea* cultures. Duramycin (1 mg/mL) was used as a control antibiotic. All experiments were performed in biological and technical triplicates.

### 4.6. Biosensor Assays: Luciferase Assays in LB-Liquid Medium

The potential modes of action of the produced antimicrobial compounds were determined using whole-cell *B. subtilis* biosensor strains (TMB1617; Table 3) harboring pBS3C*lux* derivates. Overnight cultures of the biosensors were cultivated in LB supplemented with the corresponding antibiotics for selection. Day cultures were inoculated 1:250 without antibiotics in fresh LB medium and grown to an OD_600_ of 0.3–0.4. Cells were subsequently diluted to an OD_600_ of 0.05 and incubated in a 96-microtiter well plate (95 µL per well; black walls, clear bottom, Greiner Bio-One, Frickenhausen, Germany) at 37 °C with shaking using a Synergy HTX plate reader (BioTek Instruments GmbH, Bad Friedrichshall, Germany). Luminescence and OD_600_ were measured in 5-min intervals. After one hour of incubation, the biosensors were induced with 5 μL of the sterile supernatants (2.5–5% (*v*/*v*)) and positive controls (15 µg/mL bacitracin zinc salt and duramycin [0.5 µg/mL] for TMB1617 and nisin [10 µg/mL] for TMB2120; the concentrations were selected based on the minimum inhibitory concentrations (MICs) reported in the relevant literature) [39,71]. Thus, supernatants in concentrations of 5% (*v*/*v*) of plate reader wells for days one to six, or 2.5% (*v*/*v*) of plate reader wells for days eight to fourteen, were found to have no inhibitory effect on the growth of the biosensor strains. The microplate was subsequently placed back in the microplate reader to continue luminescence and OD_600_ measurements in the aforementioned intervals for 11 h. Quantification of luminescence was achieved by calculating relative luminescence units (RLU) defined as the luminescence divided by the OD_600_ at a given time point. Biosensor induction was visualized by plotting RLU as a function of time using GraphPad Prism (version 5, San Diego, CA, USA). Experiments were performed in biological and technical triplicates.

### 4.7. Identification of Natural Resistance Mechanisms in B. subtilis W168

During the plate-based biosensor overlay assays, naturally resistant single *B. subtilis* colonies growing in the zone of inhibition were isolated and verified regarding resistance development via repeat overlay assays (Table A1). After repeated rounds of selection, the highly resistant spontaneous mutant strain (NRS-3) and the original strain (TMB5845) were subjected to whole-genome sequencing. Isolation of genomic DNA from the selected strains was performed using the MACHEREY-NAGEL NucleoSpin^®^ Microbial DNA kit (Düren, Germany). Strains were sequenced by the sequencing service of the LMU Biozentrum (Martinsried, Germany) using Illumina 1.9 paired-end and Oxford Nanopore sequencing methods. Genome alignment and analysis for the *B. subtilis* strains were performed using the open-source platform Galaxy EU (24.1). The quality of Illumina paired-end data was verified using FastQC (Galaxy v.0.11.8) [77]. To automate quality and adapter trimming, the sequencing data were analyzed with Trim Galore! (Galaxy v.0.4.3.1; threshold: 20; maximum error rate: 0.1; minimum reads length: 20) and sequences bearing an average quality below the threshold value were subsequently removed using Trimomatic (Galaxy v.0.36.5; Average quality required (20); Number of bases to average across (4)) [78]. High-quality paired-end data were aligned to the genome of the reference strain *B. subtilis* 168 (RefSeq: GCF_000009045.1) [103] using the snippy tool under the default parameters (Galaxy Version 3.2) [36]. The assembled data were visualized and single-nucleotide polymorphisms and insertions/deletions were localized using the JBrowse genome browser (Galaxy Version 1.16.11 + galaxy1) [99]. Identified mutated genes were amplified from gDNA of the naturally resistant *B. subtilis* strains using Q5^®^ polymerase and the corresponding oligonucleotide pairs (TM7677/TM7678 for *pssA*; TM7679/TM7680 for *yhbD*; TM0059/TM0062 for *mdtR*) and verified by sequencing. Effects of SNPs on transcription and translation were cross-checked using CLC Main Workbench 7 (Qiagen, Hilden, Germany).

### 4.8. Generation of B. subtilis W168 Loss-of-Function Mutants

The *B. subtilis* loss-of-function mutants were generated to verify the mutations responsible for resistance. For the PCR-based fusion of antibiotic resistance cassettes with long flanking homology regions, the kanamycin (*kan*^R^) and spectinomycin (*spec*^R^) resistance cassettes were respectively amplified from vectors pDG783 and pDG1726 with the corresponding oligonucleotide pairs TM0137/TM0138 and TM0141/TM0142. The up-stream and down-stream DNA fragments (~1 kb) of target genes were amplified from isolated gDNA of *B. subtilis* W168 (for *yhbD*: TM7752/TM7753 and TM7754/TM7755; for *mdtR*: TM7748/TM7749 and TM7750/TM7751). For DNA amplification via PCR, Q5^®^ polymerase was used. Extensions of 24–26 nucleotides (nt) that were complementary to the 5′ and 3′ ends of the amplified marker cassette were added to the 5′ end of the reverse and forward primers of the respective front and back flanking regions. Transformation of *B. subtilis* strains was performed as previously described using 100 µL of gDNA or 50 µL of DNA amplification via PCR [73]. For gDNA isolation, 800 µL of overnight culture was mixed with 800 µL of SC buffer (0.15 M NaCl, and 0.01 M C_6_H_5_Na_3_O_7_) and harvested by centrifugation (13,000 rcf, 1 min). The cell pellet was resuspended in 1 mL SC buffer and 20 µL lysozyme (15 mg/mL) and incubated at 37 °C for 15 min. The reaction was stopped by adding 1 mL of 1 M NaCl, and the crude gDNA was filtered (0.45 µm). Successful deletion of the genes in the *B. subtilis* genome was confirmed via colony PCR using OneTaq^®^ polymerase and the specific primers pairs (TM0059/TM0062 and TM0058/TM0062 for *mdtR::spec*^R^; TM7679/TM7680 and TM7679/TM0147 for *yhbD*::*kan*^R^; TM7677/TM7678 and TM0057/TM7678 for *pssA*::*mls*^R^).

## Figures and Tables

**Table 1:** Genome analysis for *A. coerulea* strains.

	*A. coerulea* TMS085	*A. coerulea* DSM 43675
GC Content [%]	72.55	72.59
Plasmids	0	0
Genome Length	8,188,457 bp	8,190,213 bp
CDS	7908	7883
Repeat Regions	104	104
tRNA	64	65
rRNA	15	15
Number of genes for:		
metabolism	972	962
DNA processing	105	104
cell envelope	21	20
regulation and cell signaling	59	58
cellular processes	88	92
membrane transport	43	54

**Table 2:** Oligonucleotides used in this work.

Oligonucleotide	Sequence 5′-3′
**TM7677 (*pssA*_fwd)**	CGTCAATCAACGCGTCTTGTC
**TM7678 (*pssA*_rev)**	CCGCTATGAGCTGCTGAACTAATTC
**TM7679 (*yhbD*_fwd)**	GCATGACCGCTGATGAATTGCTTC
**TM7680 (*yhbD*_rev)**	CACAATCTGCTTCCATGACAGC
**TM0059 (*mdtR*_fwd)**	GTGCGGATCAGTTAATGTCC
**TM0062 (*mdtR*_rev)**	ATTCCGCACAAAGCAGAAGC
**TM7748 (*mdtR*_up_fwd)**	CTTGCATTCCTCTGCGACTTGG
**TM7749 (*mdtR*_up_rev)**	CCTATCACCTCAAATGGTTCGCTGCGGACATTAACTGATCCGCAC
**TM7750 (*mdtR*_do_fwd)**	CGAGCGCCTACGAGGAATTTGTATCGCTCGCACAAGCAGCAGAAACG
**TM7751 (*mdtR*_do_rev)**	CTCGTGTCGAGATCCAATGTC
**TM7752 (*yhbD*_up_fwd)**	CTCAGTGTCTTCATGCAGGAG
**TM7753 (*yhbD*_up_rev)**	CCTATCACCTCAAATGGTTCGCTGGTCCAGCTCCTTTGATGGGTAC
**TM7754 (*yhbD*_do_fwd)**	CGAGCGCCTACGAGGAATTTGTATCGCGCAAGCTCGGTGTCACAAC
**TM7755 (*yhbD*_do_rev)**	GAGCACACGCAGCCGCTCAG
**TM0147 (*kan*^R^_check_rev)**	CTGCCTCCTCATCCTCTTCATCC
**TM0058 (*spec*^R^_check_fwd)**	GTTATCTTGGAGAGAATATTGAATGGAC
**TM0057 (*mls*^R^_check_fwd)**	CCTTAAAACATGCAGGAATTGACG
**TM0141 (*spec*^R^_fwd)**	CAGCGAACCATTTGAGGTGATAGGGACTGGCTCGCTAATAACGTAACGTGACTGGCAAGAG
**TM0142 (*spec*^R^_rev)**	CGATACAAATTCCTCGTAGGCGCTCGGCGTAGCGAGGCAAGGGTTTATTGTTTTCTAAAATCTG
**TM0137 (*kan*^R^_fwd)**	CAGCGAACCATTTGAGGTGATAGG
**TM0138 (*kan*^R^_fwd)**	CGATACAAATTCCTCGTAGGCGCTCGG

**Table 3:** Whole-cell *B. subtilis*-based biosensor strains used and their corresponding antibiotics used as positive controls.

Strain	Description	Antibiotics (Positive Control)
**TMB1617**	P*_liaI_*-*luxABCDE*	bacitracin zinc salt (10 mg/mL)
**TMB1619**	P*_bceA_*-*luxABCDE*	bacitracin zinc salt (10 mg/mL)
**TMB2120**	P*_psdA_*-*luxABCDE*	nisin (40 mg/mL)
**TMB5600**	P*_yfiLMN231_*-*luxABCDE*	amphotericin B (250 µg/mL)
**TMB5611**	P*_blaZ_*-*luxABCDE*	penicillin G (10 µg/mL)
**TMB5831**	P*_yrzI_*-*luxABCDE*	erythromycin (100 µg/mL)
**TMB5845**	P*_helD_*-*luxABCDE*	rifampicin (100 µg/mL)

## Data Availability

The manuscript contains original data. Additional data can be found at https://doi.org/10.5281/zenodo.18288795.

## Supplementary Materials

The following supporting information can be downloaded at: https://doi.org/10.5281/zenodo.18288795, Dataset S1: Genome of *A. coerulea* TMS085; Dataset S2: proteome-comparison; Dataset S3: NRS_alignment; Dataset S4: coerumycin_alignment.

## Abbreviations

The following abbreviations are used in this manuscript:

ATCC	American Type Culture Collection
CDS	Coding Sequence
DEL	Nucleotide deletion
ED	Evolutionary distance
GBDP	Genome BLAST Distance Phylogeny
GC	Guanine plus cytosine
INS	Nucleotide insertions
*luxABCDE*	Bacterial luciferase gene cluster
MIC	Minimum inhibitory concentration
MLSA	Multilocus sequence analysis
MNP	Multiple-nucleotide polymorphisms
NC	Negative control
NCBI	National Center for Biotechnology Information
NRPS	Non-ribosomal peptide synthetase
NRS	Natural resistance strain
OD_600_	Optical density at wavelength 600 nm
PC	Positive control
PE	Phosphatidylethanolamine
PKS1	Type 1 polyketide synthases
RiPP	Ribosomally-synthesized and post-translationally modified peptide
RLU	Relative luminescence units
SNP	Single-nucleotide polymorphism
T(1/2/3)PKS	Type I/II/III polyketide synthase
TYGS	Type Strain Genome Server
*v*/*v*	Volume concentration (volume per volume)
*w*/*v*	Mass concentration (weight per volume)
w/o	without
ZOI	Zone of inhibition

## Appendix A

**Table A1:** Strains and plasmids used in this work.

Strain	Relevant Genotype/Comments	Reference
**TMS085**	*Actinomadura coerulea* Isolated from dried *S. suricatta* feces samples	This study
***Escherichia coli*** **K12**	F- lambda- *ilvG- rfb*-50 *rph-1*	Laboratory stock
***Bacillus subtilis***		
**W168**	Wild-type strain, *trpC2*	Laboratory stock
**TMB1617**	W168 *sacA*::P*_liaI_*-*luxABCDE*, *cat*^R^	[39,104]
**TMB1619**	W168 *sacA*::P*_bceA_*-*luxABCDE*, *cat*^R^	[40,41]
**TMB2120**	W168 *sacA*::P*_psdA_*-*luxABCDE*, *cat^R^*	[40,41]
**TMB5600**	W168 *ΔyfiLMN*, *sacA*::P*_yfiLMN231_*-*luxABCDE*, *cat*^R^	[102]
**TMB5611**	W168 *penP*::*kan*R *thrC*::pBS4S-P*_lepA_*_*blaI lacA*::pBS2E-P*_veg_*_*blaR1* pBS3C-P*_blaZ_*-*luxABCDE*, *cat*^R^	[105]
**TMB5831**	W168 *sacA*::P*_yrzI_*-*luxABCDE*, *cat*^R^	Laboratory stock
**TMB5845**	W168 *sacA*::P*_helD_*-*luxABCDE*, *cat*^R^	[106]
**BSU02270**	168 *pssA*::*mls*^R^	[107]
**TMB6652**	W168 *yhbD*::*kan*^R^	This work
**TMB6653**	W168 *mdtR*::*spec*^R^	This work
**TMB7270**	W168 *mdtR*::*spec*^R^*, yhbD*::*kan*^R^	This work
**TMB6654**	BSU02270 *mdtR*::*spec*^R^	This work
**TMB6655**	BSU02270 *mdtR*::*spec*^R^*, yhbD*::*kan*^R^	This work
**TMB6656**	BSU02270 *mdtR*::*spec*^R^*, yhbD*::*kan*^R^	This work
**TMB7304**	TMB1617 *pssA*::*mls*^R^	This work
**NRS-1**	TMB5845 *pssA* (C209A; T70K)	This work
**NRS-2**	TMB5845 *pssA* (C209A; T70K) *mdtR* (397_402delATATT)	This work
**NRS-3**	TMB5845 *pssA* (C209A; T70K) *mdtR* (397_402delATATT) *yhbD* (A660T; K221*)	This work
**NRS-4**	TMB1617 *pssA* (361_362insGC)	This work
***Penicillium chrysogenum***	Wild-type strain	Laboratory stock
***Mucor racemosus***	Wild-type strain, facultatively pathogenic (BSL-2)	Laboratory stock
***Rhizopus microspores***	Wild-type strain, facultatively pathogenic (BSL-2)	Laboratory stock
***Aspergillus niger***	Wild-type strain, facultatively pathogenic (BSL-2)	Laboratory stock
***Scopulariopsis brevicaulis***	Wild-type strain, facultatively pathogenic (BSL-2)	Laboratory stock
***Bacillus mycoides*** **ATCC 6462**	Wild-type strain, facultatively pathogenic (BSL-2)	Laboratory stock
***Pseudomonas aeruginosa*** **ATCC 27853**	Wild-type strain, facultatively pathogenic (BSL-2)	Laboratory stock
***Proteus mirabilis***	Wild-type strain, facultatively pathogenic (BSL-2)	Laboratory stock
***Staphylococcus aureus*** **ATCC 25923**	Wild-type strain, facultatively pathogenic (BSL-2)	Laboratory stock
***Cryptococcus neoformans*** **ATCC 90112**	Wild-type strain, facultatively pathogenic (BSL-2)	Laboratory stock
***Exophiala dermatitidis***	Wild-type strain, facultatively pathogenic (BSL-2)	Laboratory stock
***Candida tropicalis***	Wild-type strain, facultatively pathogenic (BSL-2)	Laboratory stock
***Candida guilliermondii***	Wild-type strain, facultatively pathogenic (BSL-2)	Laboratory stock
	Plasmids	
**pDG783**	pSB118, *kan*^R^	[108]
**pDG1726**	pSB119, *spec*^R^	[108]

*cat***^R^**, chloramphenicol resistance gene; *kan*^R^, kanamycin resistance gene; *spec*^R^, spectinomycin resistance gene; *mls*^R^, erythromycin-induced resistance gene to macrolid-, linkosamid- and streptogramin B- antibiotics.

**Table A2:** Summary of antiSMASH results for isolated *A. coerulea* TMS085.

Region	Type	From [bp]	To [bp]	Most Similar Known Cluster	Similarity
1	terpene	542,288	563,307	chlortetracycline	8%
2	RiPP-like	636,123	646,932		
3	ectoine	912,493	922,888	ectoine	100%
4	betalactone	989,986	1,030,768	fengycin	20%
5	T2PKS	1,093,539	1,166,153	WS79089B/benaphthamycin/WS79089D/WS79089A/WS79089C	10%
6	NRP-metallophore, NRPS	1,539,998	1,612,840	madurastatin D1/madurastatin D2/(−)-madurastatin C1	51%
7	other, T1PKS, oligosaccharide	1,667,958	1,742,691	calicheamicin	50%
8	hydrogen-cyanide	1,847,532	1,860,418	aborycin	14%
9	betalactone	2,465,733	2,495,379		
10	betalactone	2,542,380	2,572,520	desulfoclethramycin/clethramycin	8%
11	hydrogen-cyanide	2,690,977	2,703,833	SF2487	4%
12	lanthipeptide-class-iii, NRPS	3,165,819	3,218,527	coprisamide C/coprisamide D	26%
13	terpene, lanthipeptide-class-iv	3,493,758	3,532,807	hopene	46%
14	T1PKS, *hglE*-KS	3,661,908	3,712,709	hexacosalactone A	4%
15	PKS-like	4,359,738	4,400,775	vazabitide A	15%
16	terpene	4,461,851	4,483,998	oxalomycin B	9%
17	terpene	4,722,511	4,748,510	hopene	46%
18	lanthipeptide-class-ii	5,564,955	5,588,149	cinnamycin B	26%
19	terpene	5,615,419	5,636,324	isorenieratene	28%
20	T1PKS	5,730,292	5,776,768	maklamicin	4%
21	T3PKS	6,811,507	6,852,568	lagunapyrone A/lagunapyrone B/lagunapyrone C	22%
22	terpene	7,785,299	7,806,492	2-methylisoborneol	50%
23	NI-siderophore	7,865,176	7,896,360	peucechelin	20%

BGCs exhibiting a similarity of greater than 20% to known gene clusters and unknown BGCs (gray). The gene clusters responsible for the synthesis of antifungal, anticancer, and antibacterial substances are denoted by the colors green, yellow, and blue, respectively.
